# Glyceryl triacetate promotes blood–brain barrier recovery after ischemic stroke through lipogenesis-mediated IL-33 in mice

**DOI:** 10.1186/s12974-023-02942-3

**Published:** 2023-11-15

**Authors:** Haidong Wei, Luming Zhen, Shiquan Wang, Liufei Yang, Shuyue Zhang, Yuanyuan Zhang, Pengyu Jia, Tianyue Wang, Kui Wang, Yan Zhang, Lei Ma, Jianrui Lv, Pengbo Zhang

**Affiliations:** 1 Department of Anesthesiology, The Second Affiliated Hospital of Xi’an Jiaotong University, Xi’an, 710004 Shaanxi China; 2 Department of Anesthesiology and Perioperative Medicine, Xijing Hospital, The Fourth Military Medical University, Xi’an, 710032 Shaanxi China; 3 Department of Anesthesiology, The First Affiliated Hospital of Xi’an Jiaotong University, Xi’an, 710061 Shaanxi China

## Abstract

**Background:**

Lipid metabolism has a crucial role in neural repair in neurodegenerative diseases. We recently revealed that lipogenesis-mediated interleukin-33 (IL-33) upregulation lead to blood–brain barrier (BBB) repair after ischemic stroke. However, manipulating the key enzyme fatty acid synthase (FASN) to enhance lipogenesis was very challenging. Glyceryl triacetate (GTA) was used as a donor of acetate and precursor of acetyl coenzyme A, the key substrate for de novo lipogenesis catalyzed by FASN. Therefore, we hypothesized that GTA would promote lipogenesis the peri-infarct after ischemic stroke and contribute to the BBB repair through IL-33.

**Methods:**

Middle cerebral artery occlusion (MCAO) was performed on C57BL mice and GTA was gavage administrated (4 g/kg) on day 2 and 4 after MCAO. Lipogenesis was evaluated by assessment of the protein level of FASN, lipid droplets, and fatty acid products through liquid chromatography-mass spectrometry in the peri-infarct area on day 3 after MCAO, respectively. BBB permeability was determined by extravasation of Evans blue, IgG and dextran, and levels of tight junction proteins in the peri-infarct area on day 7 after MCAO, respectively. Infarct size and neurological defects were assessed on day 7 after MCAO. Brain atrophy on day 30 and long-term sensorimotor abilities after MCAO were analyzed as well. The inhibitor of FASN, C75 and the virus-delivered FASN shRNA were used to evaluate the role of FASN-driven lipogenesis in GTA-improved BBB repair. Finally, the therapeutic potential of recombinant IL-33 on BBB repair and neurological recovery was evaluated.

**Results:**

We found that treatment with GTA increased the lipogenesis as evidenced by lipid droplets level and lauric acid content, but not the FASN protein level. Treatment with GTA increased the IL-33 level in the peri-infarct area and decreased the BBB permeability after MCAO. However, infarct size and neurological defect score were unchanged on day 7 after MCAO, while the long-term recovery of sensorimotor function and brain atrophy were improved by GTA. Inhibition of lipogenesis using C75 or FASN shRNA reversed the beneficial effect of GTA. Finally, exogenous IL-33 improved BBB repair and long-term functional recovery after stroke.

**Conclusion:**

Collectively, we concluded that treatment with GTA improved the BBB repair and functional recovery after ischemic stroke, probably by the enhancement of lipogenesis and IL-33 expression.

**Graphical Abstract:**

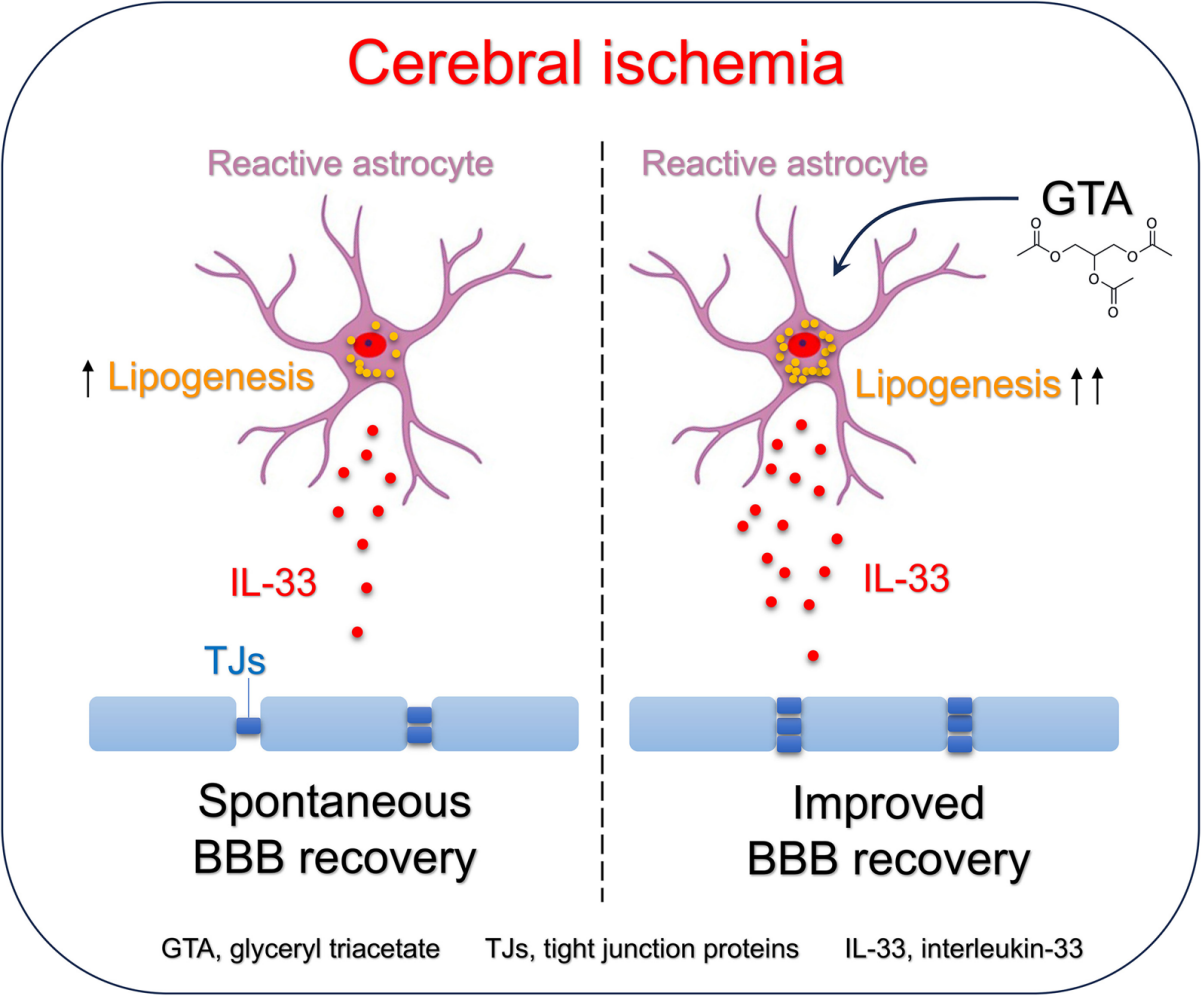

**Supplementary Information:**

The online version contains supplementary material available at 10.1186/s12974-023-02942-3.

## Introduction

Disabilities after stroke seriously compromised the life quality of the survivors [1]. Functional recovery is key for stroke-affected population to return into the family and society [2]. Clinical investigations emphasized on the critical period after the acute injury when the spontaneous recovery occurs [3, 4]. Laboratory experiments suggested that during the critical period, the microenvironment sets up the basis for the histological repair and neural plasticity which eventually rewire the destroyed neural connections [5]. Keeping a favorable microenvironment with a recovered blood brain barrier (BBB) is an essential prerequisite for neural repair as it not only keeps local blood flow and nutrition supply, but also prevents leukocytes infiltration and blood components extravasation [6]. Fortunately, we recently discovered that lipogenesis in astrocytes was essential to the spontaneous recovery of BBB after experimental ischemic stroke [7], which lead us to the comprehensive understanding of lipids metabolism in central nervous system.

Lipids are fundamental to central nervous system, not only composing all membranes, but also regulating signaling transduction, and supplying energy [8]. Besides these physiological roles in maintaining brain homeostasis and influencing neuronal functions, lipids also have important reparative roles in neurodegenerative diseases [8–10]. In addition to being associated with the occurrence of ischemic stroke [11–14], circulating lipids could also contribute to predicting the outcome of ischemic stroke [15–18]. Genetic variations of ATP binding cassette transporters ABCA1 and apolipoprotein E could imply the etiology of ischemic stroke [19, 20]. In animal studies, brain lipids were significantly altered after cerebral reperfusion, both spatially and temporally [21–23]. It was showed that targeting autotaxin to reduce lysophosphatidic acid or targeting other bioactive lipids could ameliorated the acute injury of cerebral ischemia [24–26]. The neural reparative role of lipids after acute cerebral ischemia wasn’t uncovered until recently when it was reported that polyunsaturated fatty acids promoted neurovascular restoration and short-chain fatty acids improved brain connectivity and blood–brain barrier function after cerebral ischemia [27–29]. Nevertheless, the role of brain intrinsic lipids alterations in ischemic stroke recovery was not fully understood. Although we discovered that lipogenesis-upregulated interleukin-33 (IL-33) expression in astrocytes in the peri-infarct lead to BBB repair after stroke [7], manipulating the key driving enzyme fatty acid synthase (FASN) to enhance lipogenesis was very challenging, as the activator of FASN has been lacking. Meanwhile, it is very difficult to increase the protein level of FASN by in vivo overexpression due to the large size mRNA sequence. Considering the basic elements of biochemical reactions, the way by substrate may help to increase lipogenesis. The availability of acetyl-coenzyme A is key because it is not only one of the two starter substrates of de novo lipogenesis, it is also the substrate of malonyl-CoA, the other stater substrate of de novo lipogenesis [30]. Glyceryl triacetate (GTA), an acetate donor, was proven to increase acetyl-CoA in brain [31]. Therefore, we hypothesized that GTA could promote lipogenesis in astrocytes and improve BBB repair after cerebral ischemia, and FASN mediated IL-33 upregulation might be involved.

## Methods

### Animals

C57BL/6 mice were provided by the Experimental Animal Center of Xi’an Jiaotong University. Mice were housed in a condition of 22 ± 1 °C air temperature, humidity of 50 ± 1% and 12/12 h light/dark cycle. The inclusion criteria for middle cerebral artery occlusion (MCAO) were male, aged from 8 to10 weeks and weighed from 22 to 25 g. Mice used for primary astrocytes separation were postnatal 1 day old. A total of 436 mice including 5 neonatal mice were used in the experiment. The experimental design and protocols were all complied to the National Institutes of Health Guide for the Care and Use of Laboratory Animals (NIH Publications No. 80-23), which were also approved by the Animal Care and Use Committee of Xi’an Jiaotong University. A total of 431 mice were subjected to MCAO surgery or sham operation, and 143 were excluded because of failure of MCAO surgery or death after MCAO.

### Mouse cerebral ischemia and reperfusion injury

We only used male mice in this study because estrogen has a significant impact on central nervous system. Before surgery, mice were fed freely with food and tap water. As described previously [7], the right middle cerebral artery was transient occluded in mice. Briefly, mice received MCAO under isoflurane anesthesia with inhaled concentration of 1.5–2%. After anesthesia, a silicon-coated suture (RWD Life Science) was placed through the right external carotid artery and advanced into the internal carotid artery to block the blood flow into middle cerebral artery. The suture was removed to allow reperfusion after 60 min ischemia. The rectal temperature of mice was monitored and maintained at 37 ± 0.5 °C by using a heating pad. The blood flow of the ischemic area was monitored by a laser Doppler system and the probe was placed on the defined spot of the skull (2 mm caudal and 4 mm lateral to the bregma). An 80% decrease and 70% recovery of the local cerebral blood flow was regarded as a successful MCAO surgery. Sham operation was performed only without insertion of the suture.

### Drug treatment and adeno-associated virus injection

GTA from Sigma-Aldrich (90240, Germany) was orally delivered on day 2 and 4 after MCAO. The dosage was 4 g/kg body weight of mice according to a previous report [31]. C75, a specific inhibitor of FASN from MedChemExpress (HY-12364, China) and prepared in normal saline with the solubilizers of 5% DMSO, 40% PEG300 and 5% Tween-80. The concentration of C75 was 1 mg/ml. The C75 was administrated intraperitoneally with a dosage 10 mg/kg body weight 72 h after MCAO as we used before [7]. Vehicle control contained the corresponding solubilizers in normal saline. Mouse recombinant IL-33 (rIL-33) from Biolegend (580502, CA, USA) was prepared in normal saline to a concentration of 0.2 mg/ml and administrated into the lateral cerebral ventricle 72 h after MCAO as we used before [7]. Equal volume of normal saline was used as vehicle control of rIL-33. The astrocytes-specific adeno-associated virus (AAV) 2/9 encoding for FASN-shRNA-EGFP or scrambled RNA (scRNA)-EGFP with GFAP promoter was constructed by BrainVTA Co., Ltd. (Wuhan, China). The shRNA sequence was: 5’-CCGGCGTCTATACCACTGCTTACTACTCGAGTAGTAAGCAGTGGTATAGACGTTTTTG-3’. The virus injection was stereotactically guided into the peri-infarcted area 4 weeks before MCAO surgery. The amount of AAV was 200 nl (titer 2 × 10^12^ vg/ml) per coordinate. And the stereotactic parameters were AP 0.3 mm, ML 2.0 mm, DV 1.8 mm from bregma for cortex, and AP 0.3 mm, ML 2.0 mm, DV 3.00 mm from bregma for striatum. The knockdown efficiency of the shRNA was confirmed as we reported [7].

### Neurological function assessment

#### Neurological defects evaluation

A neurobehavior assessment based on Garcia was performed on day 7 after MCAO [32]. The observer was unaware of the experimental grouping. The whole system consists of 6 tests with multiple evaluations of sensory and motor function based on spontaneous activity, symmetrical movements of the upper and lower limbs, forepaw outstretching, climbing, body proprioception and response to vibrissal touch.

#### The forelimb grip strength test

The long-term forepaw grip strength was assessed after MCAO. Briefly, the maximum grip strength was gauged using a strength meter (BIOSEB, BIO-GS3, France). A T-shaped metal rod was connected to the meter. The mouse was gently suspended by the tail. Then, the forepaws of mice were symmetrically allowed to grip both transverse tips of the T-shaped rod. The mouse body was maintained in a horizontally position and then pulled away from the rod until it let go. The maximum strength would be automatically recorded. Three repetitions were conducted for each mouse and the average maximum strength was recorded.

#### The adhesive removal test

The adhesive removal test is reliable in evaluating sensorimotor function of mouse after cerebral ischemia [33]. This test was performed by attaching two small adhesive tape strips (0.3 cm × 0.4 cm) onto the bare part of both forepaws. The contact time (mouse began to react to the presence of the adhesive tapes: shaking paws or bringing paws to mouth) and removal time for each tape were recorded. The asymmetry magnitude of each parameter was also compared.

### Assessment of cerebral infarct size

Staining with 2,3,5-triphenyltetrazolium chloride (TTC, MP210312610, MP Biomedicals) was performed to reveal the infarcted area on day 7 after MCAO. After euthanasia with overdose sodium pentobarbital (MP Biomedicals), mouse brains were quickly removed and cut into 1-mm coronal slices on ice. The slices were immersed in 2% TTC in saline at 37 °C for 20 min. After fixation in 4% paraformaldehyde for 24 h, the images of the stained slices were acquired using a digital camera. The healthy area pixels of contralateral (VC) and ipsilateral hemisphere (VL) were calculated by using an image analysis software. The relative infarct percentage (%I) was obtained using the following formula: % I = 100 × (VC – VL)/VC.

### Evaluation of brain atrophy

Brain atrophy was assessed based on Nissl staining of cerebral slices 30 days after MCAO. The frozen sectioned mouse cerebral coronal slices (15-μm-thick) at 300-μm intervals approximately from 1.3 to − 1.7 mm to bregma were rinsed with PBS and stained in 1% methyl violet (C0117, Beyotime) for 10 min. Next, the slices were gently rinsed with water. Then, the slices were transferred into graded ethanol. After treatment with xylenes and mounted with neutral balata, the slices were scanned by SLIDEVIEW VS200 (Olympus, Japan). The relative atrophy percentage was according to the formula: (total VC area – total VL area) / total VC area × 100%.

### Evaluation of BBB disruption

#### Evans blue extravasation

Evans blue was delivered into the blood circulation of mouse through tail vein injection 6 days after MCAO. Each mouse received 4 mg Evans blue in 200 μl normal saline. The mouse was euthanized and transcardially perfused with 20 ml saline 24 h after Evans blue injection. Mouse brains were then harvested and prepared into 1-mm coronal slices. The image of extravasated Evans blue in the cerebral slices was taken by a digital camera and the ischemic hemisphere was separated. The separated hemispheres were processed with lysis buffer. A standard curve of Evans blue concentration was plotted. And the concentration of Evans blue in each sample was calculated based on the standard curve through a fluorescence spectrophotometer.

#### Dextran extravasation

Dextran-Texas Red™ 10 000 MW (D1828, Invitrogen) was intravenously delivered into the blood circulation of mouse 7 days after MCAO. Each mouse received 0.5 mg dextran in 200 μl saline. The mice were fixed with 4% paraformaldehyde 120 s after dextran administration. Mouse brains were then collected and cut into 12-μm-thick slices. After immunofluorescence staining with rabbit anti-GFAP antibody (1:500, GTX108711, Genetex, CA, USA), the images of slices were acquired using a laser confocal fluorescence microscope (Olympus FluoView FV1200). The extravasated dextran was based on its fluorescence intensity by the *ImageJ* software (National Institutes of Health, USA).

### Lipid droplets staining

Nile red staining was applied to reveal neutral lipid droplets on fixed brain slices or on primary astrocytes. Cerebral slices or cell coverslips were immunofluorescence stained with rabbit anti-GFAP antibody (1:500, GeneTex), and Alexa Fluor 647-conjugated donkey anti-rabbit secondary antibody (1:500, Jackson ImmunoResearch) was used as the secondary antibody. Then, the slices or coverslips were incubated with Nile red (1 μg/ml, HY-D0718, MedChemExpress) at room temperature for 15 min. After mounting with an antifade medium (VECTASHIELD, USA), the samples were subjected to confocal fluorescence microscope observation. LipidSpot™ 610 (1:1000, 70069T, Biotium) was also used to observe the lipid droplets in tissues or primary astrocytes. After staining of GFAP as above, the samples were stained with LipidSpot™ 610 at room temperature for 30 min. Then, the samples were mounted and observed.

### Immunofluorescence staining

Mice were fixed by transcardial perfusion with 4% paraformaldehyde. Mouse brains were removed and subjected to dehydration in 30% sucrose. The brains were then frozen and cut into coronal slices with a thickness of 12-μm. The sections were stained as we previously reported [7]. Briefly, after permeabilization and blocking with donkey serum, the samples were immunoreacted with rabbit anti-FASN antibody (1:400, ab22759, Abcam, Cambridge, London, UK), chicken anti-GFAP antibody (1:800, GTX85454, GeneTex), rabbit anti-GFAP antibody (1:400, GeneTex), goat anti-IL-33 antibody (1:500, AF3626, R&D system, USA), at 4 °C overnight, and then Alexa Fluor 594-conjugated donkey anti-rabbit secondary antibody (1:500, Invitrogen), Alexa Fluor 488-conjugated donkey anti-chicken secondary antibody (1:500, Jackson ImmunoResearch), Alexa Fluor 647-conjugated donkey anti-rabbit secondary antibody (1:500, Jackson ImmunoResearch), Alexa Fluor 594-conjugated donkey anti-goat secondary antibody (1:500, Invitrogen), Alexa Fluor 594-conjugated donkey anti-mouse secondary antibody (1:500, Invitrogen) were used as the corresponding secondary antibodies. The samples were mounted with the antifade medium and observed under a laser confocal fluorescence microscopy (Olympus FluoView FV1200).

### Western blot analysis

Mouse brains were harvested 3 or 7 days after MCAO. The tissues corresponding to the peri-infarcted areas were separated on ice. The sample proteins were collected by sonic lysis in RIPA lysis buffer (Beyotime, China) on ice. A BCA kit from Beyotime was used to determine the protein content of each sample. The denature condition was 100 °C for 2 min. Proteins samples (40 μg per lane) were electrophoresis separated by a 10% sodium dodecyl sulfate–polyacrylamide gel. The separated proteins were transferred to a polyvinylidene difluoride membrane. The membranes were then blocked with 10% skim milk at room temperature for 1 h. For immunoblotting of the membrane, the following primary antibodies were used at 4 °C overnight: rabbit anti-FASN antibody (1:1000, ab22759, Abcam), rabbit anti-ZO-1 antibody (1:1000, 21773-1-AP, Proteintech, China), rabbit anti-Occludin antibody (1:1000, 27260-1-AP, Proteintech), rabbit anti-Claudin 5 antibody (1:1000, AF5216, Affinity Biosciences, China), goat anti-IL-33 antibody (1:2000, AF3626, R&D system), rabbit anti-β-actin (1:3000, NC011, Zhuangzhibio, China). The following secondary antibodies were used to reveal the protein bands on the membrane: horseradish peroxidase-conjugated goat anti-rabbit secondary antibody (1:5000, EK020, Zhuangzhibio) and horseradish peroxidase-conjugated donkey anti-goat secondary antibody (1:5000, EK030, Zhuangzhibio). The incubating condition was room temperature for 2 h. The images of immunoblotted membranes were taken by the ChemiDoc MP imaging system (Bio-Rad, CA, USA).

### Primary astrocyte culture and oxygen–glucose deprivation

Primary astrocytes from cortex were separated from the neonatal C57BL/6 pups of 1-day old according to a previous study with slight modification [34]. Purified primary astrocytes were cultured in DMEM (Thermo Fisher Scientific) with 10% fetal bovine serum (Thermo Fisher Scientific). For oxygen–glucose deprivation (OGD), the cell culture medium was changed into glucose-free DMEM (Thermo Fisher Scientific). After that, cell plates were placed into a hermetic chamber (Billups-Rothenberg, USA) flushed and suppled with an anaerobic gas mixture of 5% CO_2_ and 95% N_2_ at 37 ºC. After 120 min of OGD, astrocytes were returned to normal culture conditions with complete culture medium and supplied with 5% CO_2_ in the air. GTA and C75 were added 24 h after OGD. The concentration was 2 mM for GTA and 10 μM for C75 [35, 36]. After 48 h, astrocytes were fixed with 4% paraformaldehyde for 15 min at room temperature or collected for protein extraction.

### Liquid chromatography-tandem mass spectrometry (LC–MS/MS)

On day 3 after MCAO, mouse was anesthetized and transcardially perfused with 20 ml saline. Then, mouse brain was removed and the peri-infarct area was quickly dissected on ice. The sample was then snap frozen in liquid nitrogen and stored at − 80 ºC until analysis. The sample was homogenized in tenfold-volume water. Then tenfold-volume methanol (containing 5 μg/ml of internal standards) and tenfold-volume chloroform was mixed. The mixture was processed by 10 min ultra-sonication. After centrifugation at 3000*g* for 10 min, 32 μl chloroform layer was evaporated to dryness. The residues were mixed with 20 μl HoBt (in DMSO), 40 μl cholamine (in DMSO with 200 mM TEA) and 20 μl HATU (in DMSO) and incubated at room temperature for 5 min. A 120 μl acetonitrile was added and followed by centrifuging at 14,000*g* for 15 min at 4 °C prior to LC–MS/MS analysis.

Target fatty acids were quantified by an Agilent 1290 Infinity II UHPLC system coupled to a 6470A Triple Quadrupole mass spectrometry (Santa Clara, CA, United States). Samples were injected onto a Waters UPLC BEH C18 column (100 mm × 2.1 mm, 1.7 μm). The mobile phase consisted of water (phase A) and acetonitrile (phase B), both with 0.1% formate. The chromatographic separation was conducted by a gradient elution program as follows: 0 min, 10% B; 4 min, 30% B; 8 min, 45% B; 11 min, 50% B; 14 min, 70% B; 15 min, 100% B; 18 min, 100% B; 18.1 min, 10% B; 20 min, 10% B. The column temperature was 40 °C. The temperatures of ESI + source drying gas was 300 °C and sheath gas was 350 °C. The flow rate of ESI + source drying gas and sheath gas were 5 and 11 L/min, respectively. The pressure of nebulizer was 45 psi, and capillary voltage was 3000 V. The dynamic multiple reaction monitoring (dMRM) was used to acquire data in optimized MRM transition (precursor—> product). The total scan time of per cycle was 500 ms. The raw data were processed by Agilent MassHunter Workstation Software (version B.08.00) and ChemStation (version E.02.02.1431).

### Statistical analysis

The Prism 8 (Graphpad Software, San Diego, California, USA) was used for statistical analysis. Neurological scores based on Garcia were presented as median with interquartile range and tested by Kruskal–Wallis method. Other data were expressed as mean with standard deviation and tested by one-way ANOVA with post hoc Tukey method. Repeated data including grip strength and adhesive removal times were processed using two-way ANOVA, and multiple comparisons were corrected by Tukey method. A *P* value less than 0.05 was considered statistically significant.

## Results

### GTA treatment enhanced lipogenesis in the peri-infarct area after ischemic stroke

**Fig. 1:**
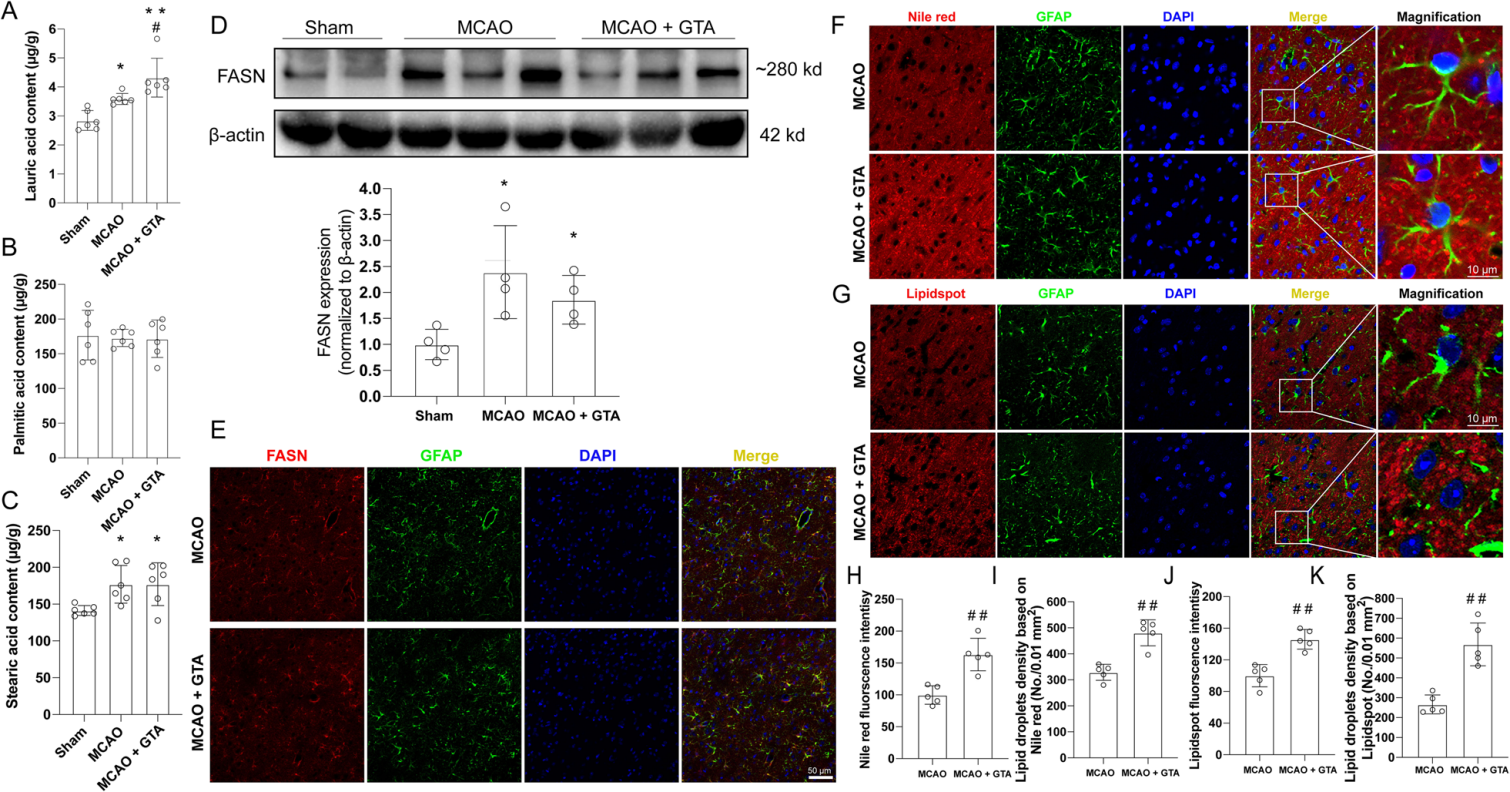
Treatment with GTA increased lipogenesis in the peri-infarct area on day 3 after cerebral ischemia. **A** Free lauric acid level in the peri-infarct area. **B** Free palmitic acid level in the peri-infarct area. **C** Free stearic acid level in the peri-infarct area. **D** The protein level of FASN in the peri-infarct area. **E** The expression of FASN in astrocytes in the peri-infarct area. **F** Nile red staining of tissue lipids in the peri-infarct area. **G** Lipidspot staining of tissue lipids in the peri-infarct area. **H** Relative Nile red fluorescence intensity. **I** Lipid droplets concentration based on Nile red (**F**). **J** Relative Lipidspot fluorescence intensity. **K** Lipid droplets concentration based on Lipidspot (**K**). *n* = 6 for **A**–**C**, *n* = 4 for **D**, *n* = 5 for **H**–**K**. Compared with Sham, *P < 0.05, **P < 0.01. Compared with MCAO, ^#^*P* < 0.05, ^##^*P* < 0.01 (tested by one-way ANOVA with post hoc Tukey method). Scale bar = 50 μm in **E**, scale bar = 10 μm in **F** and **G**

The LC–MS/MS data showed that, compared to sham group, free lauric acid, the 12-carbon intermediate product of lipogenesis was increased in the peri-infarct area on day 3 after MCAO (Fig. 1A, *P* < 0.05). And GTA supplementation further increased the content of lauric acid as compared to MCAO group (Fig. 1A,  *P* < 0.05). However, free palmitic acid, the end product of FASN-driven lipogenesis was not changed after MCAO or GTA treatment (Fig. 1B). Free stearic acid, the derivative product from palmitic acid elongation, was increased after MCAO (Fig. 1C,   *P* < 0.05), while GTA treatment had no effect on stearic acid level as compared to MCAO (Fig. 1C). The FASN was also assessed by western blotting and immunofluorescence staining. FASN was increased 3 days after MCAO (Fig. 1 D, 1E, *P* < 0.05). However, GTA treatment had no effect on FASN level as compared to MCAO group. The staining of lipid droplets using Nile red and Lipidspot™ 610 was shown in Fig. 1F and G. Relative quantification of lipids content based on the fluorescence intensity of Nile red (Fig. 1H, *P* < 0.01) or Lipidspot™ 610 (Fig. 1J, *P* < 0.01) showed that GTA treatment increased the lipid content in the peri-infarct area compared to MCAO. The concentration of lipid droplets as indicated by of Nile red (Fig. 1I, *P* < 0.01) or Lipidspot™ 610 (Fig. 1K, *P* < 0.01) was also increased after GTA treatment compared to MCAO.

### GTA treatment increased IL-33 expression in the peri-infarct area after ischemic stroke

**Fig. 2:**
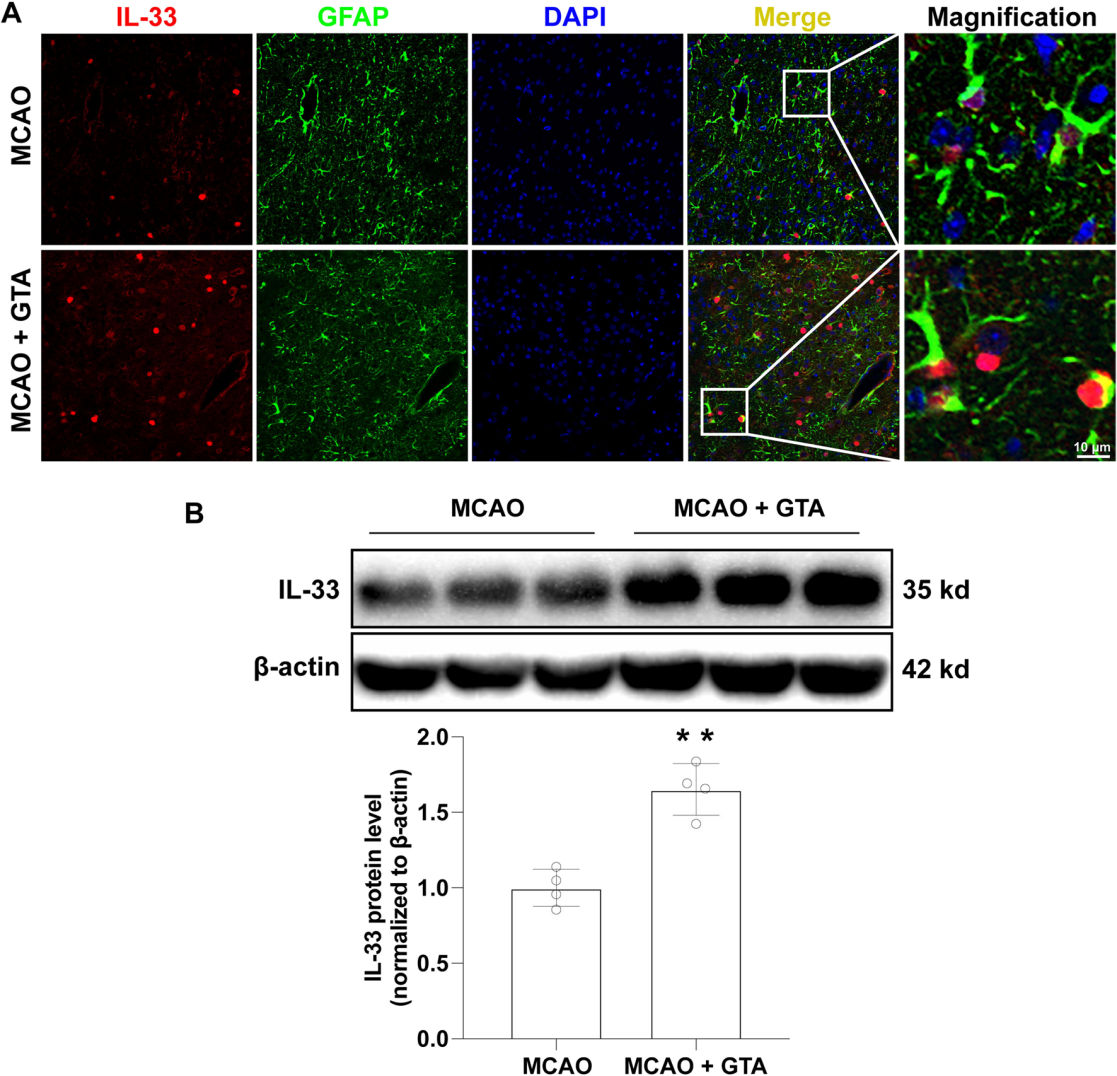
Treatment with GTA increased IL-33 expression in astrocytes in the peri-infarct area on day 3 after cerebral ischemia. **A** The expression of IL-33 in astrocytes in the peri-infarct area. **B** The protein level of IL-33 by western blotting. *n* = 4, compared with MCAO, **P < 0.01 (tested by Student *t*-test). Scale bar = 10 μm

IL-33 was evaluated by immunofluorescence staining and western blotting in the peri-infarct area on day 3 after reperfusion. The reactive astrocytes were indicated with highly expressed GFAP and hypertrophy of cell body (Fig. 2A), which identified the peri-infarct area after MCAO. IL-33 was co-localized with the nucleus of reactive astrocyte and GTA treatment increased IL-33 expression significantly as compared to MCAO (Fig. 2A). The protein quantification data showed that GTA treatment upregulated IL-33 expression as compare to MCAO group (Fig. 2B, *P* < 0.01).

### GTA treatment improved BBB recovery on day 7 after reperfusion

**Fig. 3:**
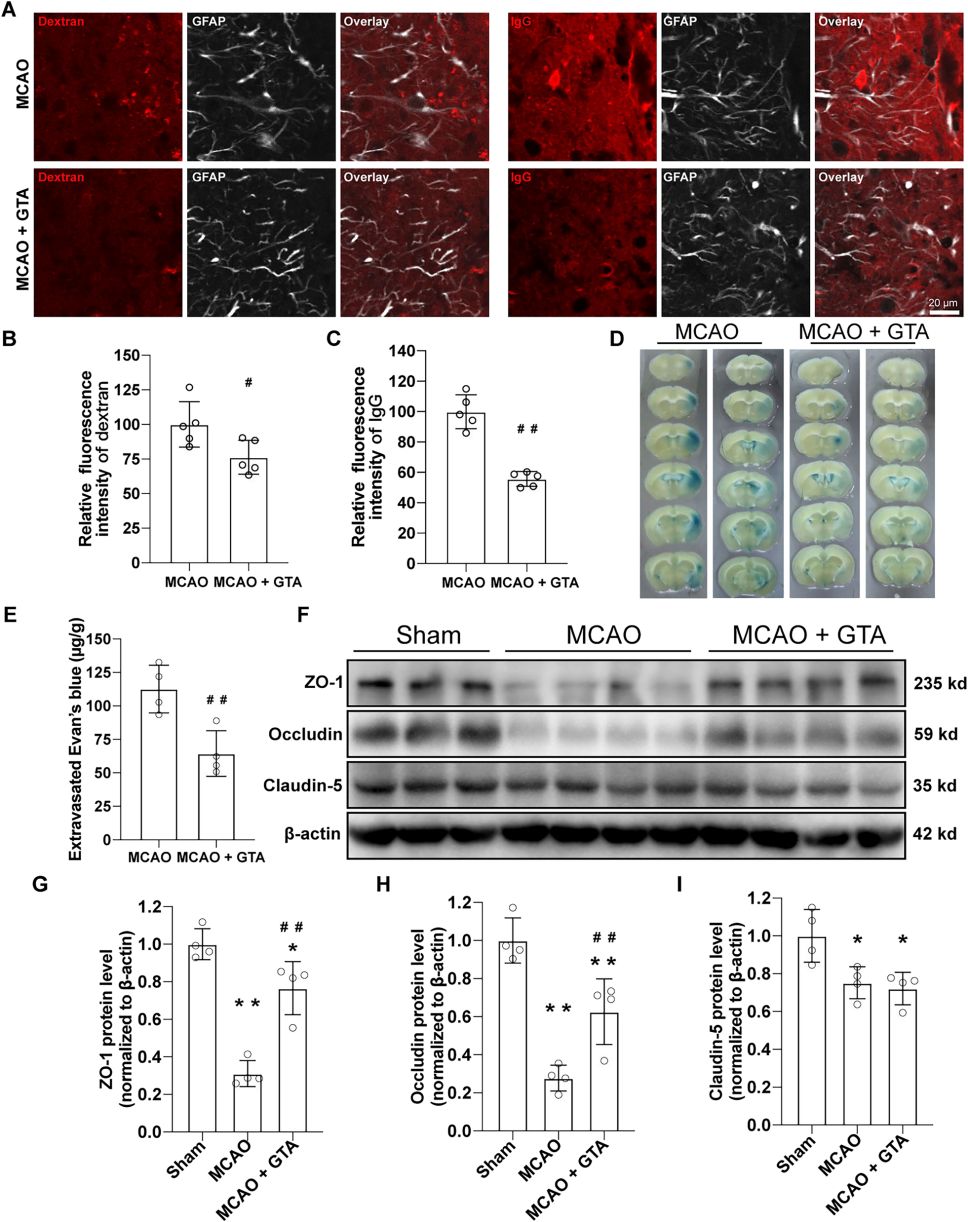
Treatment with GTA improved BBB permeability in the peri-infarct area on day 7 after cerebral ischemia. **A** The representative image of extravagated dextran and IgG in the peri-infarct area. **B** and **C** Quantification of dextran and IgG intensity based on **A**. **D** The representative image of Evans blue leakage. **E** Quantification of **D**. **F**, Western blotting of tight junction proteins in the peri-infarct area. **G**–**I** Quantification of ZO-1, Occludin and Claudin-5 based on **F**. *n* = 5 for **B** and **C**, *n* = 4 for **E**–**I**. Compared with Sham, *P < 0.05, **P < 0.01. Compared with MCAO, ^#^*P* < 0.05, ^##^*P* < 0.01 (tested by one-way ANOVA with post hoc Tukey method). Scale bar = 20 μm

The BBB leakage was assessed with different molecular indicators, 10 kd dextran, autologous IgG and Evans blue dye (Fig. 3A–E). Relative quantification data showed that GTA treatment prevented the extravagation of both 10 kd dextran (Fig. 3B, *P* < 0.05) and IgG (Fig. 3C, *P* < 0.01) in the peri-infarct area as compared to MCAO group. GTA treatment also prevented the leakage of Evans blue dye to the injured hemisphere compared with MCAO group (Fig. 3E, *P* < 0.01). The tight junction proteins ZO-1, Occludin and Claudin-5 in the peri-infarct area were detected by western blotting (Fig. 3F) on day 7 after MCAO. Quantitative analysis showed that, after MCAO, all tight junction proteins were decreased (Fig. 3G–I, *P* < 0.05), however, GTA treatment increased the protein level of ZO-1 (Fig. 3G, *P* < 0.01) and Occludin (Fig. 3H, *P* < 0.01) but Claudin-5 (Fig. 3I), as compared to MCAO.

### GTA treatment improved the functional recovery after cerebral ischemia

**Fig. 4:**
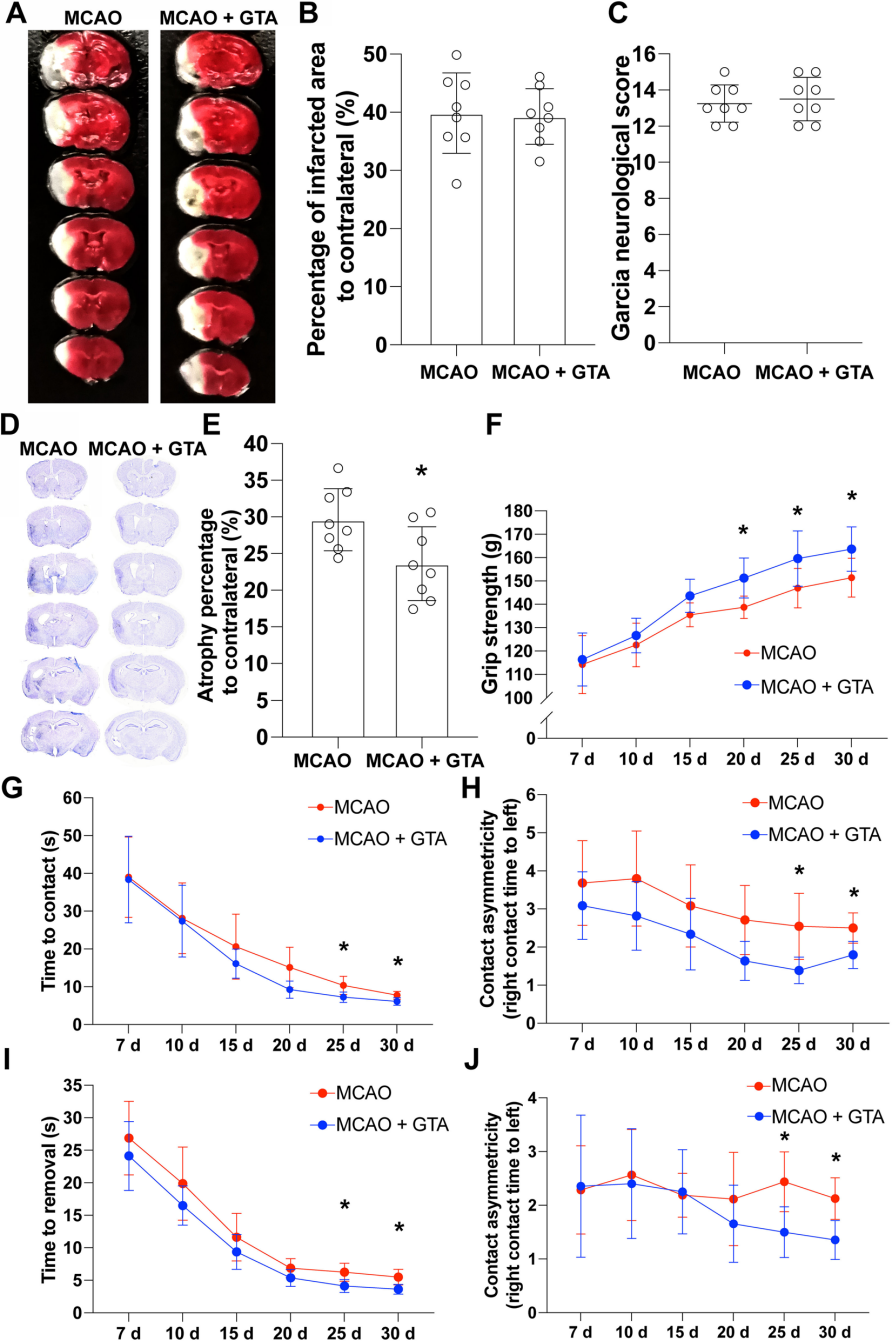
Treatment with GTA improved long-term neurological function after cerebral ischemia. **A** The representative image of TTC staining for infarct size on day 7 after cerebral ischemia. **B** Quantification of relative infarct size on day 7 after cerebral ischemia. **C** The neurological defect score on day 7 after cerebral ischemia. **D** Nissl staining on day 30 after cerebral ischemia. **E** Brain atrophy analysis based on **D**. **F** The recovery of forepaw strength over 30 days after cerebral ischemia. **G** and **H** Contact time and contact asymmetry over 30 days after cerebral ischemia. **I** and **J**, Removal time and removal asymmetry over 30 days after cerebral ischemia. *n* = 8, compared with MCAO, *P < 0.05, **P < 0.01. **B** and **E** were analyzed by Student *t*-test, **C** was analyzed by Kruskal–Wallis test, **F**–**J** were analyzed by two-way ANOVA and multiple comparisons corrected with Tukey method

The ischemic injury was assessed by TTC staining and neurological defect score on day 7 after reperfusion. Representative infarction by TTC staining was shown in Fig. 4A. Compared to MCAO group, GTA treatment had no effect on either the infarct size (Fig. 4B) or the neurological defect score (Fig. 4C). Long-term recovery was assessed by cerebral atrophy and sensorimotor function. Representative Nissl staining on day 30 after MCAO was shown in Fig. 4D. Compared to MCAO group, GTA treatment improved the brain atrophy percentage (Fig. 4E, *P* < 0.05). The forepaw grip strength recovery was improved by GTA treatment on day 20, 25 and 30 after MCAO (Fig. 4F, *P* < 0.05). The recovery of the sensory function of left palm was improved by GTA treatment on day 25 and 30 after cerebral ischemia as compared to MCAO, indicated by contact time (Fig. 4G, *P* < 0.05) and contact asymmetry (Fig. 4H, *P* < 0.05), respectively. The left forelimb motor function recovery was also improved by GTA treatment on day 25 and 30 after cerebral ischemia as compared to MCAO, indicated by removal time (Fig. 4I, *P* < 0.05) and removal asymmetry (Fig. 4J, *P* < 0.05), respectively.

### Inhibition of FASN reversed the effect of GTA treatment on lipogenesis in the peri-infarct area

**Fig. 5:**
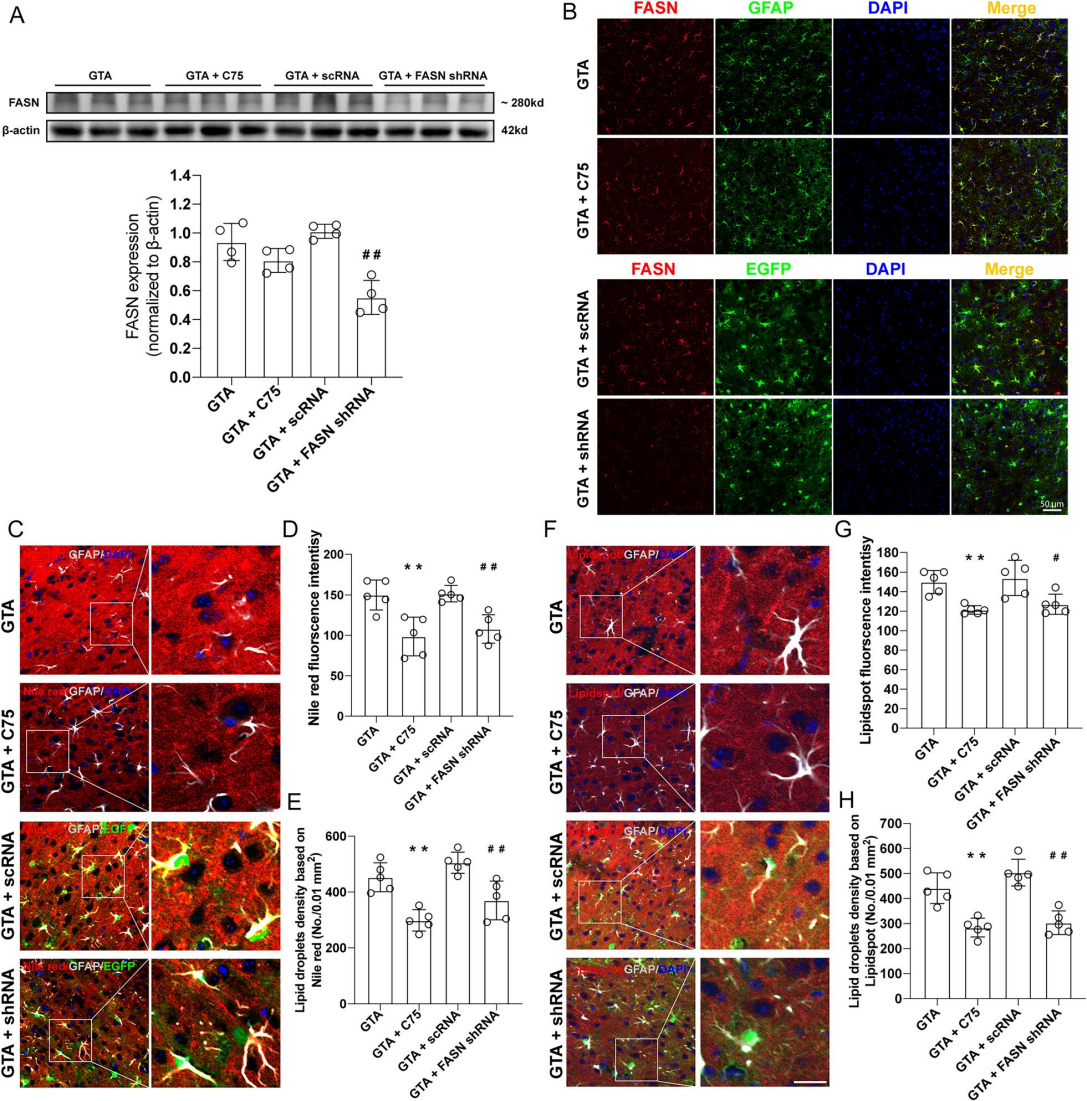
FASN inhibition reversed GTA-promoted lipogenesis in the peri-infarct area on day 3 after cerebral ischemia. **A** The protein level of FASN in the peri-infarct area. **B** The expression of FASN in astrocytes in the peri-infarct area. **C** Nile red staining of tissue lipids in the peri-infarct area. **D** Lipidspot staining of tissue lipids in the peri-infarct area. **E** Relative Nile red fluorescence intensity. **F** Lipid droplets concentration based on Nile red (**C**). **G** Relative Lipidspot fluorescence intensity. **H** Lipid droplets concentration based on Lipidspot (**D**). *n* = 4 for **A**, *n* = 5 for **C**–**H**. Compared with GTA, **P < 0.01. Compared with GTA + scRNA, ^#^*P* < 0.05, ^##^*P* < 0.01 (tested by one-way ANOVA with post hoc Tukey method). FASN shRNA with EGFP was expressed under GFAP promoter. Scale bar = 50 μm

FASN expression was assessed by western blotting and immunofluorescence staining in presence of its specific inhibitor C75 or shRNA 3 days after MCAO. Data showed that after cerebral ischemia and GTA treatment, C75 didn’t affect the protein level of FASN (Fig. 5 A, B), while FASN shRNA decreased the protein level of FASN in astrocytes in the peri-infarct area (Fig. 5 A, B, *P* < 0.01). The staining of neutral lipid droplets using Nile red and Lipidspot™ 610 was shown in Fig. 5C and F. Relative quantification of lipids content based on the fluorescence intensity of Nile red (Fig. 5D, *P* < 0.01) or Lipidspot™ 610 (Fig. 5G, *P* < 0.01) showed that C75 reversed the increase of lipid content in the peri-infarct area after GTA treatment. Similarly, FASN shRNA also reversed the increase of lipid content in the peri-infarct area as compared to GTA + scRNA group, based on the fluorescence intensity of Nile red (Fig. 5D, *P* < 0.01) or Lipidspot™ 610 (Fig. 5G, *P* < 0.05). The concentration of lipid droplets as indicated by of Nile red (Fig. 5E, *P* < 0.01) or Lipidspot™ 610 (Fig. 5H, *P* < 0.01) was also decreased after C75 or FASN shRNA treatment compared to GTA treatment, respectively.

### Inhibition of FASN reversed the increase of IL-33 induced by GTA treatment after cerebral ischemia

**Fig. 6:**
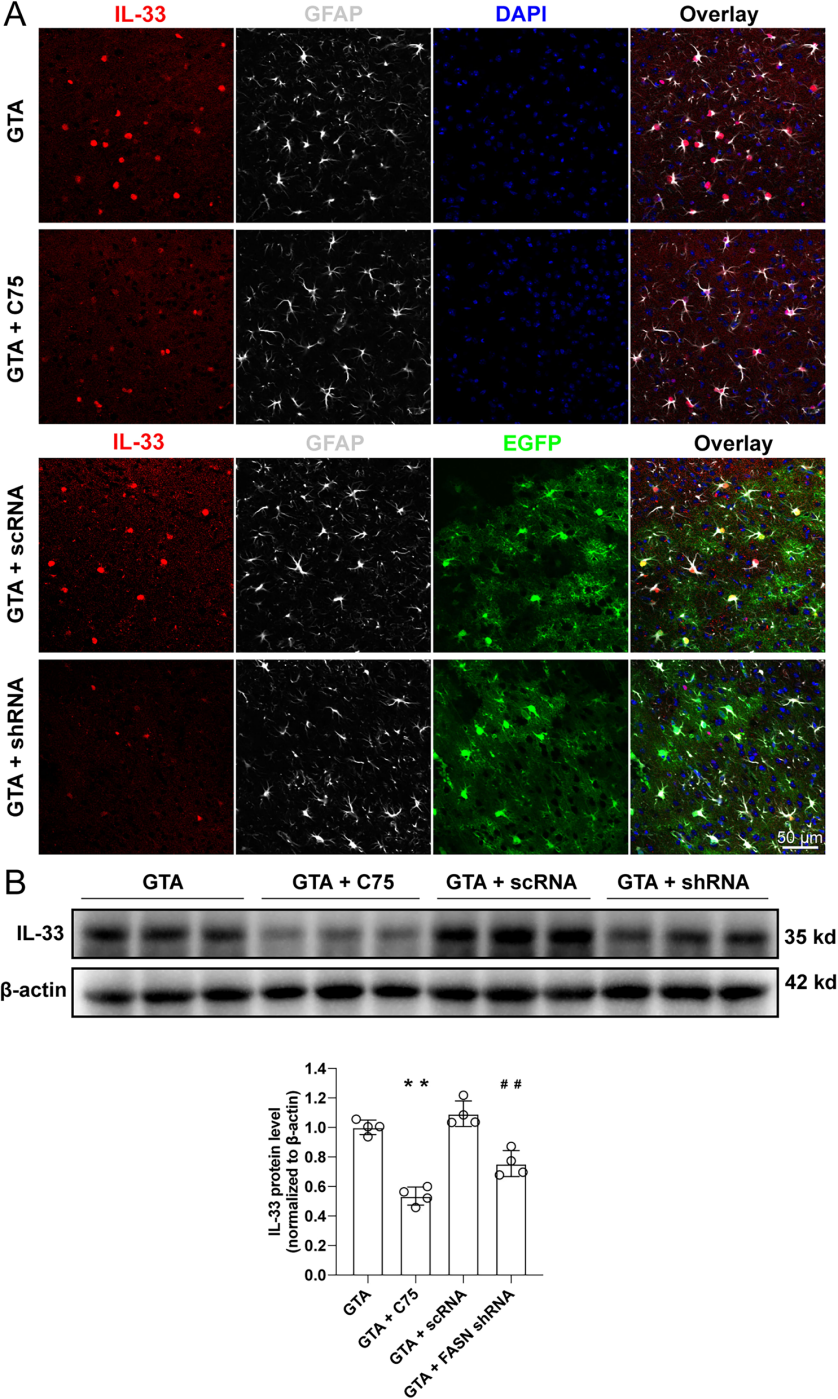
Inhibition of lipogenesis reversed GTA-induced IL-33 upregulation in the peri-infarct area on day 3 after cerebral ischemia. **A** The expression of IL-33 in astrocytes in the peri-infarct area. **B** The protein level of IL-33 in the peri-infarct area by western blotting. *n* = 4, compared with GTA, **P < 0.01. Compared with GTA + scRNA, ^##^*P* < 0.01 (tested by one-way ANOVA with post hoc Tukey method). FASN shRNA with EGFP was expressed under GFAP promoter. Scale bar = 50 μm

By immunofluorescence staining of the peri-infarct area on day 3 after reperfusion, we revealed that both C75 and FASN shRNA reversed the upregulation of IL-33 induced by GTA treatment in reactive astrocytes (Fig. 6A). The protein quantification data showed that both C75 and FASN shRNA reversed the increase of IL-33 protein level induced by GTA treatment in the peri-infarct area (Fig. 6B, *P* < 0.01).

### Inhibition of FASN reversed GTA-improved BBB permeability after cerebral ischemia

**Fig. 7:**
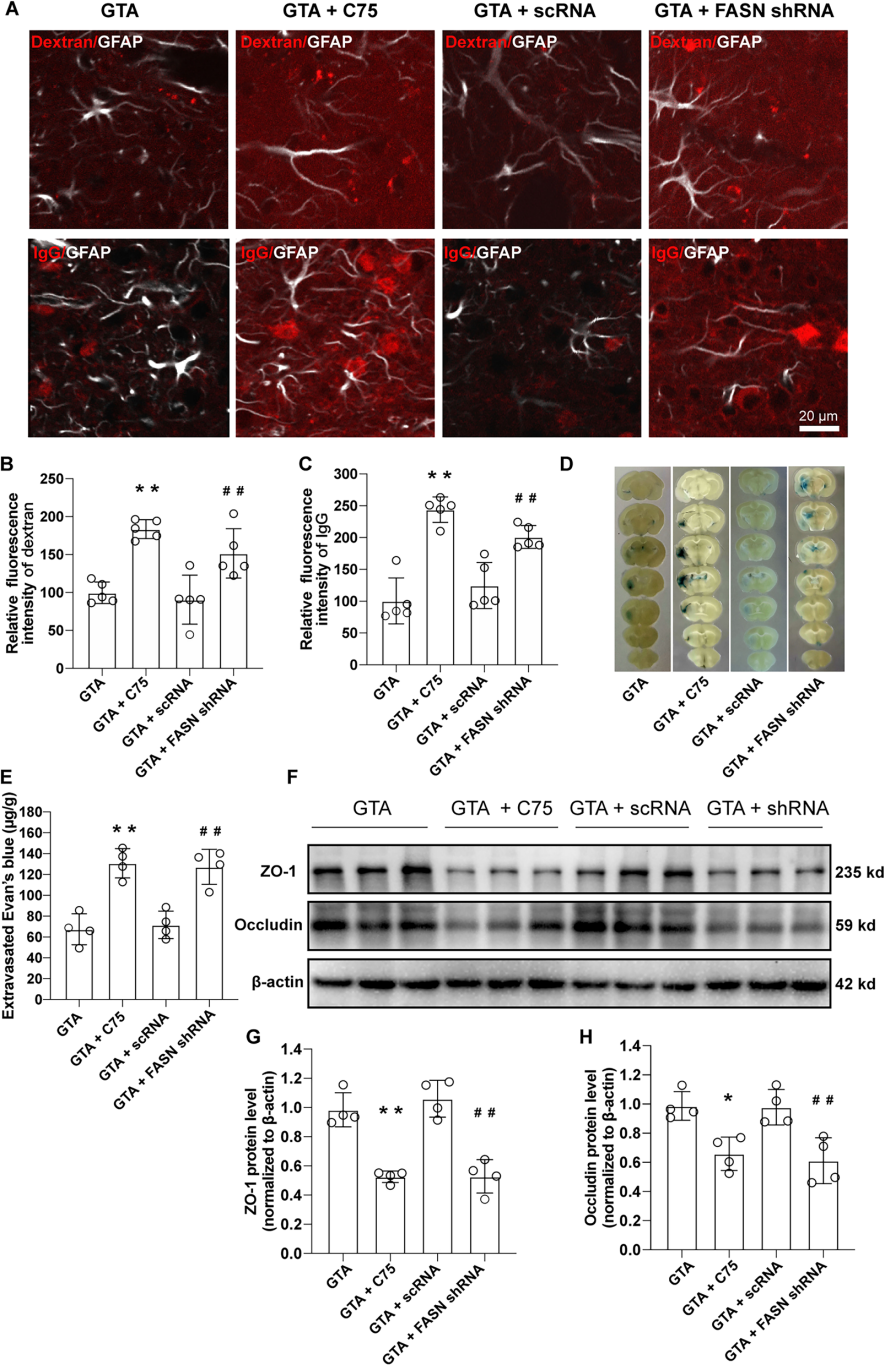
Inhibition of lipogenesis reversed GTA-induced improvement of BBB permeability in the peri-infarct area on day 7 after cerebral ischemia. **A** The representative image of extravagated dextran and IgG in the peri-infarct area. **B** and **C** Quantification of dextran and IgG intensity based on **A**. **D** The representative image of Evans blue leakage. **E** Quantification of **D**. **F** Western blotting of tight junction proteins. **G** and **H** Quantification of ZO-1 and Occludin based on **F**. *n* = 5 for **B** and **C**, *n* = 4 for **E**–**I**. Compared with GTA, *P < 0.05, **P < 0.01. Compared with GTA + scRNA, ^##^*P* < 0.01 (all tested by one-way ANOVA with post hoc Tukey method). FASN shRNA was expressed under GFAP promoter. Scale bar = 20 μm

Inhibition of FASN-driven lipogenesis using C75 or astrocyte-specific FASN shRNA hampered the improvement of BBB leakage as indicated by 10 kd dextran, autologous IgG, and Evans blue dye (Fig. 7A–E). Relative quantification data showed that C75 or FASN shRNA increased the extravagation of both 10 kd dextran (Fig. 7B, *P* < 0.01) and IgG (Fig. 7C, *P* < 0.01) in the peri-infarct area as compared to GTA or GTA + scRNA group, respectively. C75 or FASN shRNA also increased the leakage of Evans blue dye to the injured hemisphere compared with GTA or GTA + scRNA group, respectively (Fig. 7E, *P* < 0.01). The tight junction proteins ZO-1 and Occludin in the peri-infarct area were detected by western blotting (Fig. 7F) on day 7 after MCAO. Quantitative analysis data showed that C75 or FASN shRNA treatment decreased the protein levels of ZO-1 (Fig. 7G, *P* < 0.01) and Occludin (Fig. 7H, *P* < 0.01) compared with GTA or GTA + scRNA group, respectively.

### Inhibition of FASN hampered the improvement of functional recovery rendered by GTA treatment after cerebral ischemia

**Fig. 8:**
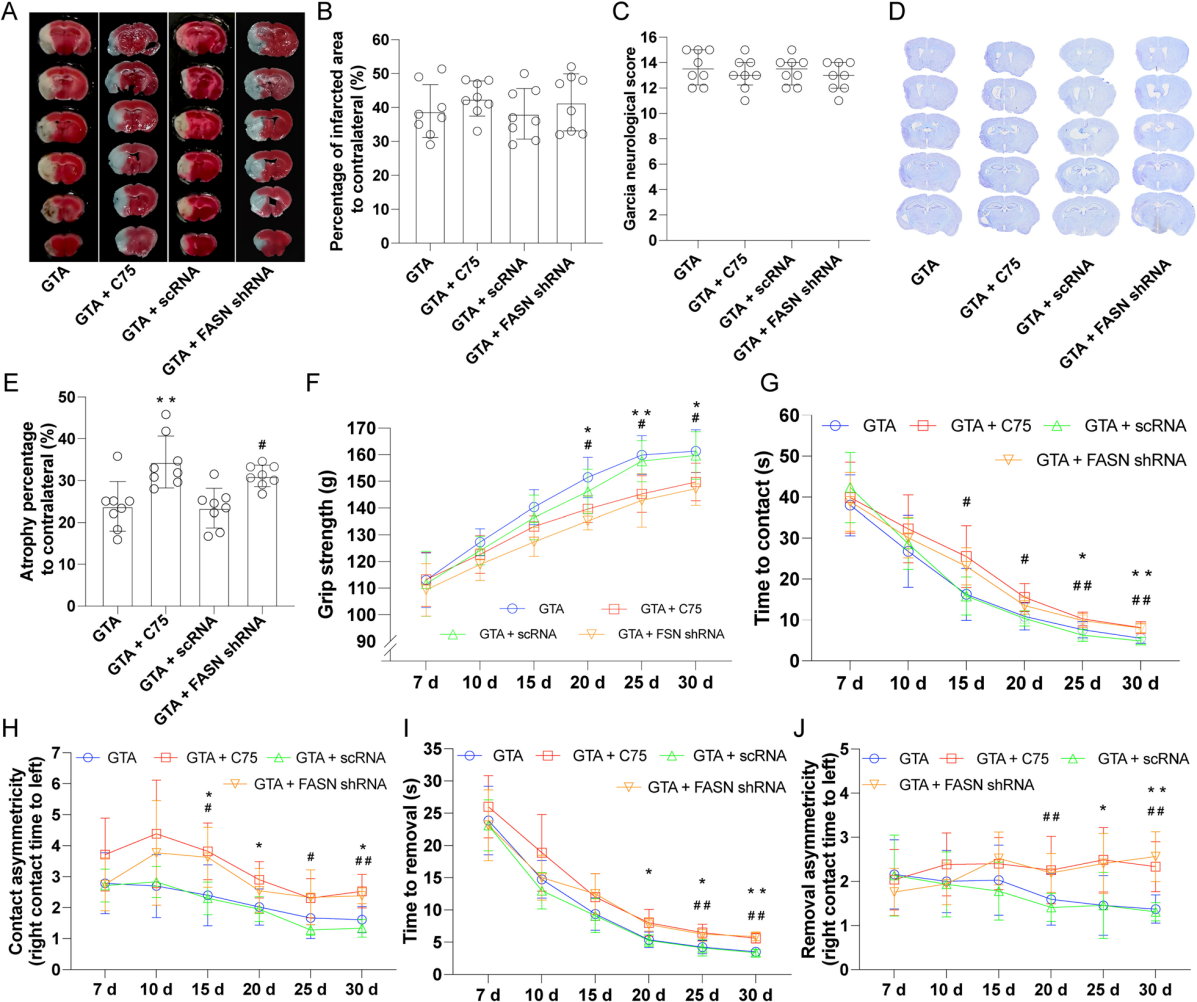
Inhibition of lipogenesis reversed GTA-induced improvement in long-term neurological function after cerebral ischemia. **A** The representative image of TTC staining for infarct size on day 7. **B** Quantification of relative infarct size. **C** The neurological defect score on day 7 after cerebral ischemia. **D** Nissl staining on day 30 after cerebral ischemia. **E** Brain atrophy analysis based on **D**. **F** The recovery of forepaw strength over 30 days after cerebral ischemia. **G** and **H** Contact time and contact asymmetry over 30 days after cerebral ischemia. **I** and **J** Removal time and removal asymmetry over 30 days after cerebral ischemia. *n* = 8, compared with GTA, *P < 0.05, **P < 0.01. Compared with GTA + scRNA, ^##^*P* < 0.05, ^##^*P* < 0.01. **B** and **E** were analyzed by one-way ANOVA with post hoc Tukey test, **C** was analyzed by Kruskal–Wallis test, **F**–**J** were analyzed by two-way ANOVA and multiple comparisons corrected with Tukey method

The infarcted area assessed by TTC staining was showed in Fig. 8A. Compared to GTA group or GTA + scRNA group, inhibition of FASN with C75 or shRNA didn’t change the infarct size on day 7 after reperfusion (Fig. 8B). The neurological defect score on day 7 didn’t show any difference among groups (Fig. 8C). Long-term histological assessment based on Nissl staining on day 30 after MCAO was shown in Fig. 8D. Compared to GTA group or GTA + scRNA group, C75 or FASN shRNA treatment increased the brain atrophy percentage (Fig. 8E, *P* < 0.05), respectively. Long-term functional analysis showed that C75 or FASN shRNA treatment decreased the forepaw grip strength on day 20, 25, and 30 after MCAO, as compared to GTA group or GTA + scRNA group (Fig. 8F, *P* < 0.05), respectively. The recovery of the left palm sensory function was deteriorated by C75 on day 25 and 30, or by FASN shRNA on day 20, 25, and 30 in presence of GTA treatment as indicated by contact time (Fig. 8G, *P* < 0.05). The contact asymmetry showed that the recovery of the left palm sensory was deteriorated by C75 treatment on day 15, 20, and 30, or by FASN shRNA treatment on day 15, 25, and 30 in presence of GTA treatment (Fig. 8H, *P* < 0.05), respectively. Moreover, the left forelimb motor function recovery was hindered by C75 or FASN shRNA treatment on day 25 and 30 compared to GTA or GTA + scRNA, indicated by removal time (Fig. 8I, *P* < 0.05) and removal asymmetry (Fig. 8J, *P* < 0.05), respectively.

### Exogenous IL-33 improved BBB recovery on day 7 after reperfusion

**Fig. 9:**
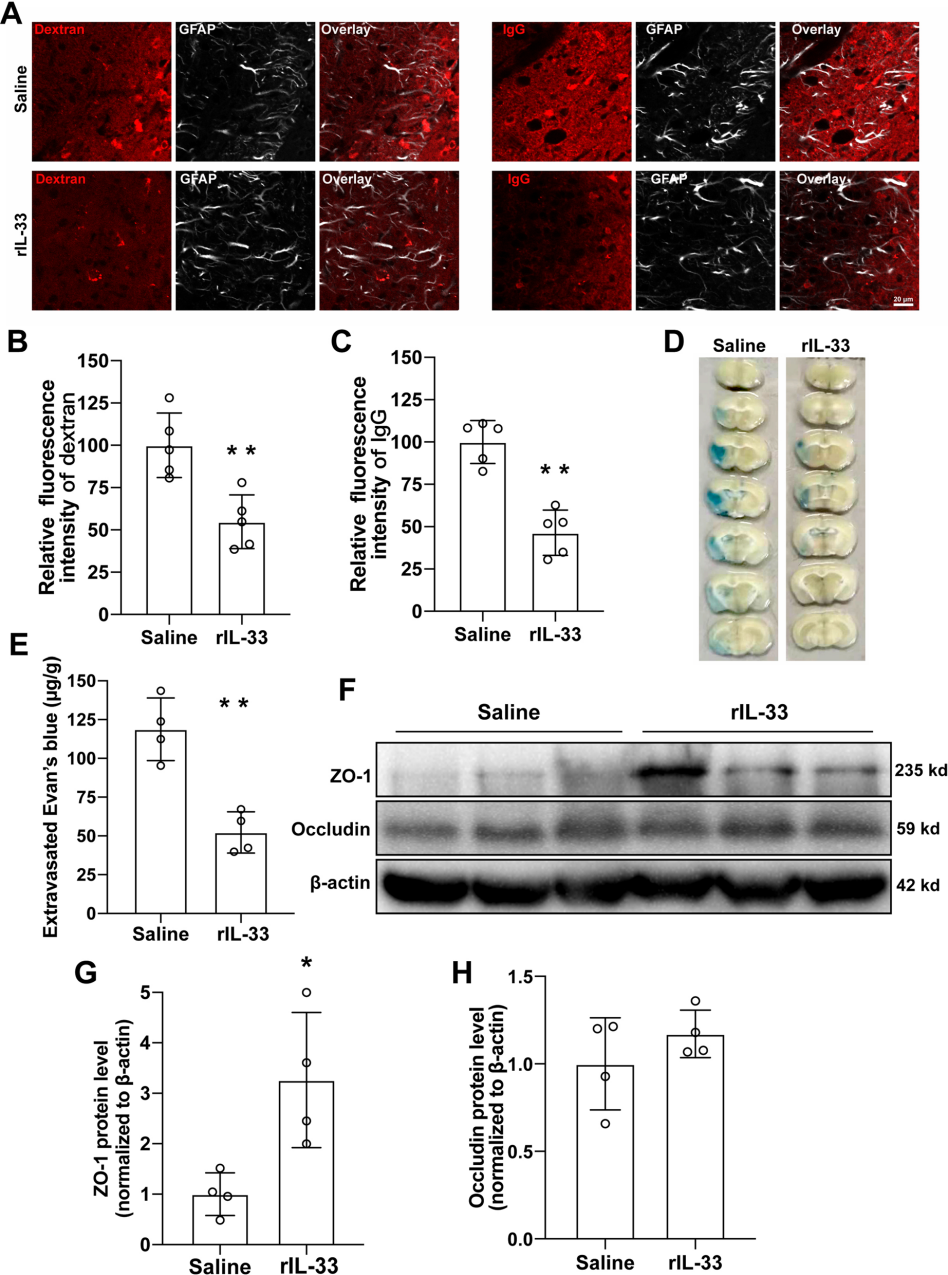
Recombinant IL-33 improved the BBB permeability in the peri-infarct area on day 7 after cerebral ischemia. **A** The representative image of extravagated dextran and IgG. **B** and **C** Quantification of dextran and IgG intensity based on **A**. **D** The representative image of Evans blue leakage. **E** Quantification of **D**. **F** Western blotting of tight junction proteins. **G** and **H** Quantification of ZO-1 and Occludin based on **F**. *n* = 5 for **B** and **C**, *n* = 4 for **E**–**I**. Compared with Saline, *P < 0.05, **P < 0.01 (tested by Student *t*-test). Scale bar = 20 μm

rIL-33 was administrated into cerebral lateral ventricle on day 3 after reperfusion. The BBB leakage was assessed as presented in Fig. 9A–E. Relative quantification showed that rIL-33 treatment improved the BBB leakage as indicated by the extravagation of both 10 kd dextran (Fig. 9B, *P* < 0.01), IgG (Fig. 9C, *P* < 0.01), and Evans blue (Fig. 9D, E, *P* < 0.01) as compared to saline treatment. The tight junction proteins ZO-1 and Occludin in the peri-infarct area were detected by western blotting on day 7 after MCAO (Fig. 9F). Quantitative analysis data showed that rIL-33 treatment increased the protein level of ZO-1 (Fig. 9G, *P* < 0.05) but not Occludin compared to saline treatment (Fig. 9H).

### Exogenous IL-33 improved the functional recovery after cerebral ischemia

**Fig. 10:**
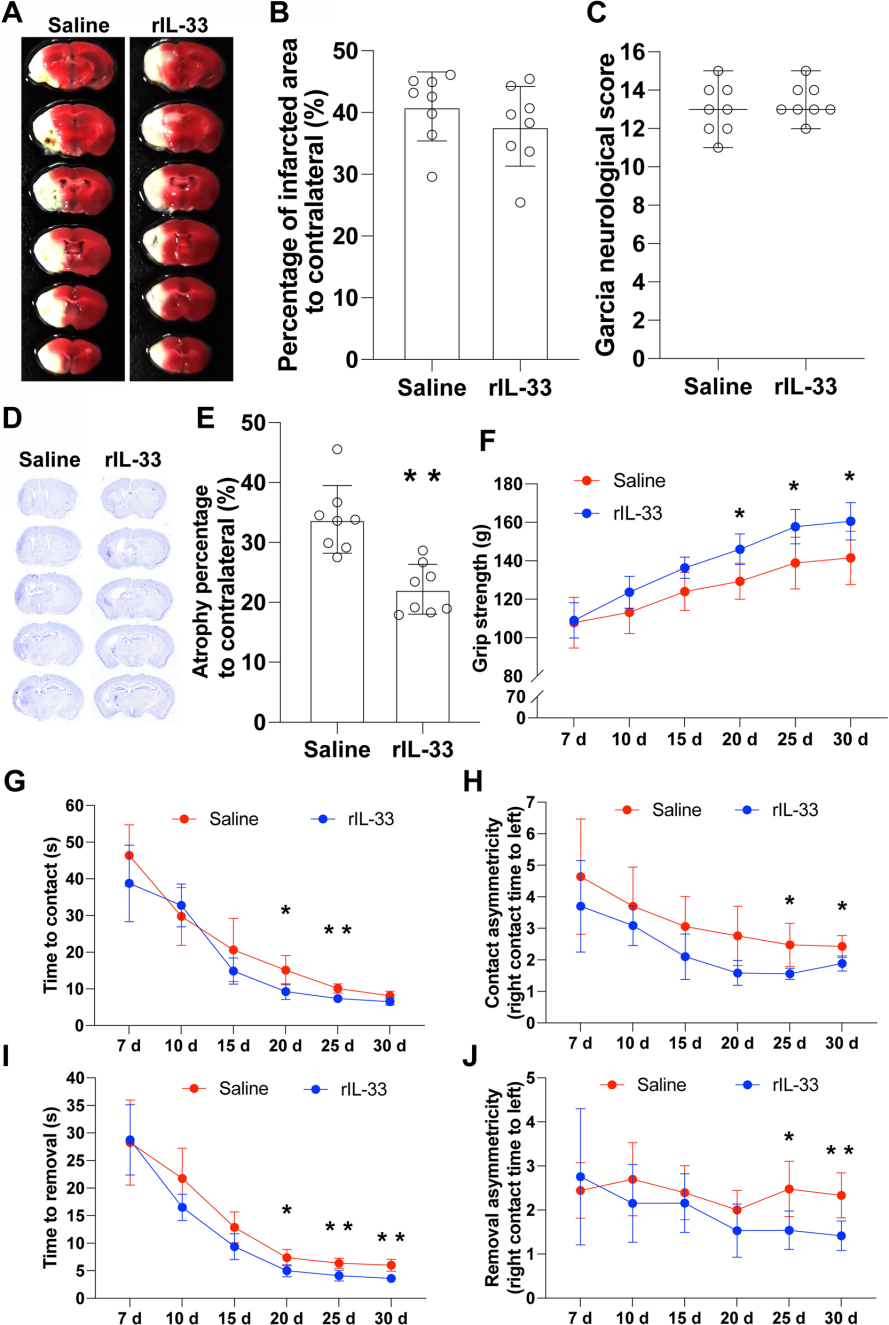
Exogenous IL-33 improved the long-term neurological function after cerebral ischemia. **A** The representative image of TTC staining for infarct size on day 7 after cerebral ischemia. **B** Quantification of relative infarct size. **C** The neurological defect score on day 7 after cerebral ischemia. **D**, Nissl staining on day 30 after cerebral ischemia. **E** Brain atrophy analysis based on **D**. **F** The recovery of forepaw strength over 30 days after cerebral ischemia. **G** and **H** Contact time and contact asymmetry over 30 days after cerebral ischemia. **I** and **J** Removal time and removal asymmetry over 30 days after cerebral ischemia. *n* = 8, compared with Saline, *P < 0.05, **P < 0.01. **B** and **E** were analyzed by Student t-test, **C** was analyzed by Student *t*-test, **F**–**J** were analyzed by two-way ANOVA and multiple comparisons corrected with Tukey method

The brain infarction on day 7 after reperfusion was shown in Fig. 10A. Compared to saline group, rIL-33 treatment had no effect on either the infarct size (Fig. 10B) or the neurological defect score (Fig. 10C). Long-term histological recovery as assessed by cerebral atrophy was shown in Fig. 10D and E. Compared to saline group, rIL-33 treatment decreased the brain atrophy percentage (Fig. 10E, *P* < 0.01). The forepaw grip strength recovery was improved by rIL-33 treatment on day 20, 25, and 30 after MCAO as compared to saline treatment (Fig. 10F, *P* < 0.05). Compared to saline treatment, the recovery of the sensory function of left palm was improved by rIL-33 treatment, indicated by contact time on day 20 and 25 (Fig. 10G, *P* < 0.05), and contact asymmetry on day 25 and 30 (Fig. 10H, *P* < 0.05), respectively. The left forelimb motor function recovery was also improved by rIL-33 treatment as compared to saline treatment, indicated by removal time on day 20, 25, and 30 (Fig. 4I, *P* < 0.05) and removal asymmetry on day 25 and 30 (Fig. 4J, *P* < 0.05), respectively.

## Discussion

In the present study, we showed that glyceryl triacetate by gavage administration increased free lauric acid in the peri-infarct area after cerebral ischemia. However, the lipogenesis enzyme FASN was not changed. GTA also increased lipid droplets level as indicated by histological methods, suggesting that lipogenesis was enhanced by GTA. We also found that IL-33, the important target molecule of FASN-driven lipogenesis, was upregulated in astrocytes by GTA. These all closely related to the improved BBB repair and neurological functional recovery after ischemic stroke. By using the specific inhibitor C75 or astrocyte-specific FASN shRNA, we revealed the role of lipogenesis in GTA-afforded IL-33 upregulation, BBB repair and neurological functional recovery. Additionally, we showed that exogenous IL-33 was effective to improve blood–brain barrier leakage and functional recovery after ischemic stroke.

The safety of GTA administration in human was demonstrated with good tolerance at high dose with no significant side effects [37]. Animal study showed that GTA could be used as a precursor of acetyl-coenzyme A in brain [31]. We found that GTA administration during the subacute phase of ischemic stroke increased the level of free lauric acid, an intermediate product for lipogenesis under FASN catalysis, indicating that GTA enhanced FASN-driven lipogenesis in the peri-infarct area after intragastric administration. The histological lipids staining with neutral lipids probes also proved this. However, the protein level of FASN was not significantly altered by GTA. This could be explained by that enzyme-driven reaction depends on multiple factors including the substrate concentration, enzyme content and activity, which is the basis that we tried to increase lipogenesis by raising the availability of acetyl-coenzyme A in brain by GTA treatment.

We previously proved that IL-33 was the mediator for spontaneous lipogenesis to improve BBB repair after stroke [7]. Here, by enhancing lipogenesis with GTA, we found that IL-33 was increased further in reactive astrocytes. It was reported that GTA exerted anti-inflammatory effect against lipopolysaccharide-induced neuroinflammation [38–40]. However, the underlying mechanism was unclear. IL-33, an important regulator for the immunological and inflammatory responses in central nerve system, has a potent role in T regulatory cells (Treg) and reparative microglia expansion after brain injury [41–44]. These findings suggest that lipogenesis-mediated IL-33 upregulation could be an important mechanism for the anti-neuroinflammation effect of GTA. However, further confirmation is still needed.

As for the improved BBB function after GTA treatment, it could be explained by the anti-inflammatory role of IL-33. Other factors could also be involved, like metabolic modulations such as histone acetylation could contribute to the anti-inflammatory effect of GTA [39]. Histone acetylation could also be the reason for increased lipogenesis with upregulation of FASN [45, 46]. However, in the present study, GTA didn’t induce the upregulation of FASN, suggesting that other factors including the substrates acetyl-coenzyme A and malonyl coenzyme A, and upstream enzymes should be accounted for the increased lipogenesis.

We analyzed the infarct size according to the BBB evaluation on day 7 after cerebral ischemia and found that the infarct size was not influenced by GTA treatment. The improved BBB function didn’t result in reduced infarct size, this could be attributed to that the brain damage has begun to turned into the repair process [5]. As a part of brain repair after stroke [6], the BBB recovery was consistent with the long-term histological and neurological outcome, as suggested by our data.

We previously found that FASN, the driving enzyme of lipogenesis, was highly expressed in reactive astrocytes after stroke [7]. In the present study, we used astrocyte-specific AAV containing FASN shRNA to blunt lipogenesis after GTA treatment. By using FASN shRNA or FASN inhibitor C75, the promotion of lipogenesis by GTA treatment and the increase of IL-33 in astrocytes were reversed, and GTA-induced improvement in BBB repair and neurological functional recovery were also reversed. These data confirmed that FASN-driven lipogenesis and lipogenesis-mediated IL-33 expression play an important role in BBB repair after stroke. By subjecting mouse primary astrocyte into OGD model, we confirmed again that FASN-driven lipogenesis was spontaneously promoted after ischemia injury in astrocytes (Additional file 1: Fig. S1A–D). Regulation of lipogenesis in astrocytes with C75 or GTA in vitro also confirmed the lipogenesis-mediated IL-33 expression (Additional file 1: Fig. S1E, F). Given the potent role of IL-33 in neuroinflammation [47], our data indicates a novel role of FASN-driven lipogenesis in brain injury and diseases besides of FASN-supported myelination and neural stem cell activation [48, 49]. We speculated that signal transducer and activator of transcription 3 (STAT3) might be involved in FASN-induced IL-33 expression, because STAT3 in astrocytes may require palmitoylation to become activated [50], and palmitic acid is synthesized under FASN catalysis. Nevertheless, the palmitoylation modification of STAT3 resulted from lipogenesis in reactive astrocytes after stroke need to be confirmed in further study.

For the therapeutic potential of IL-33, we found that single dose on day 3 after cerebral ischemia could significantly improve the BBB function and neurological recovery. The therapeutic role of IL-33 from day 5 after traumatic brain injury was also confirmed by others with intranasal administration [51]. These data provided convincing effect of IL-33 on brain function recovery after acute injury. Although microglia, Treg or other cells may conduct the reparative effect, the consistent and potent role of IL-33 in repairing injured brain after stroke is more important [41], which may have a great translational potential if the peripheral side effects could be avoided [52]. While GTA, a highly tolerated compound in human, increased local IL-33 expression in reactive astrocytes in the peri-infarct area, suggesting that it has a great translational potential and may have no significant side effects.

Some limitations exist in this study, which included that animal model only used male young mice, mice with complications like aging or diabetes have not been studied, and the specific mechanism that lipogenesis mediated IL-33 expression was unrevealed.

Collectively, we concluded that GTA improved BBB repair and functional recovery after cerebral ischemia through lipogenesis-mediated IL-33 expression in reactive astrocytes, which suggests multiple translational potentials for stroke recovery.

### Supplementary Information

**Additional file 1: Fig. S1.** Astrocyte lipogenesis and its effect on IL-33 expression at 48 h after OGD. A, The protein level of FASN by western blotting. B, The expression of FASN in primary astrocytes. C, Nile red -revealed lipid droplets in primary astrocytes. D, Lipidspot-revealed lipid droplets in primary astrocytes. E, The expression of IL-33 in primary astrocytes. F, The protein level of IL-33 in astrocytes. *n* = 4, compared with control, *P < 0.05, **P < 0.01. Compared with OGD, ^#^*P* < 0.05, ^##^*P* < 0.01 (tested by one-way ANOVA). Scale bar = 100 μm in B, scale bar = 25 μm in C, D and E.

**Additional file 2.** The uncropped gel and blot images.

## Data Availability

All data generated or analyzed during this study are included in this article and its additional information files.
