# Supplementary figures and images for: Glyceryl triacetate promotes blood–brain barrier recovery after ischemic stroke through lipogenesis-mediated IL-33 in mice
Source: J Neuroinflammation. 2023 Nov 15;20:264. doi: 10.1186/s12974-023-02942-3 (PMC10648711; doi:10.1186/s12974-023-02942-3)

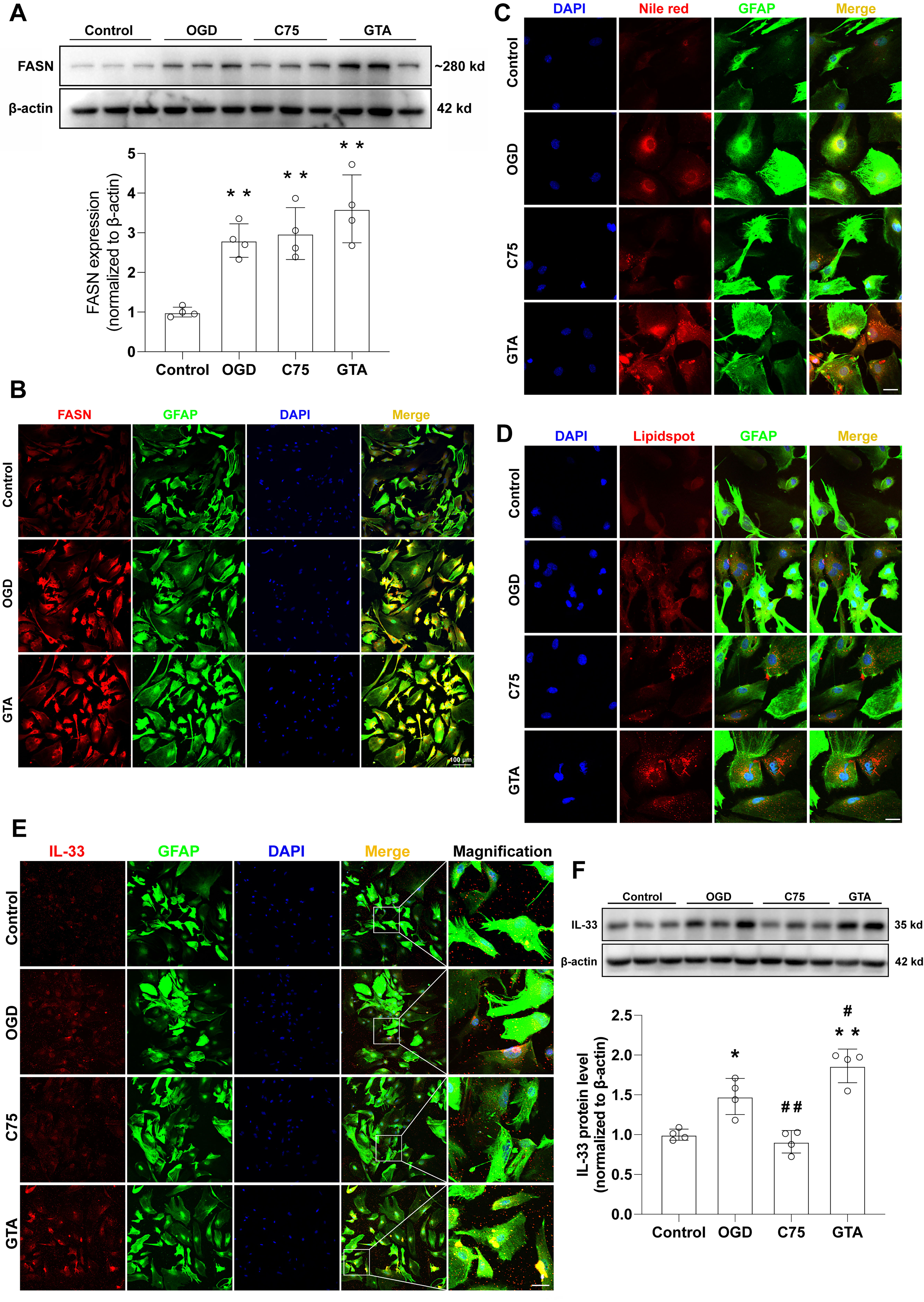

Supplement: Supplementary file 1 — Additional file 1: Fig. S1. Astrocyte lipogenesis and its effect on IL-33 expression at 48 h after OGD. A, The protein level of FASN by western blotting. B, The expression of FASN in primary astrocytes. C, Nile red -revealed lipid droplets in primary astrocytes. D, Lipidspot-revealed lipid droplets in primary astrocytes. E, The expression of IL-33 in primary astrocytes. F, The protein level of IL-33 in astrocytes. n = 4, compared with control, *P < 0.05, **P < 0.01. Compared with OGD, #P < 0.05, ##P < 0.01 (tested by one-way ANOVA). Scale bar = 100 μm in B, scale bar = 25 μm in C, D and E. [file 12974_2023_2942_MOESM1_ESM.jpg]

Figure 1D

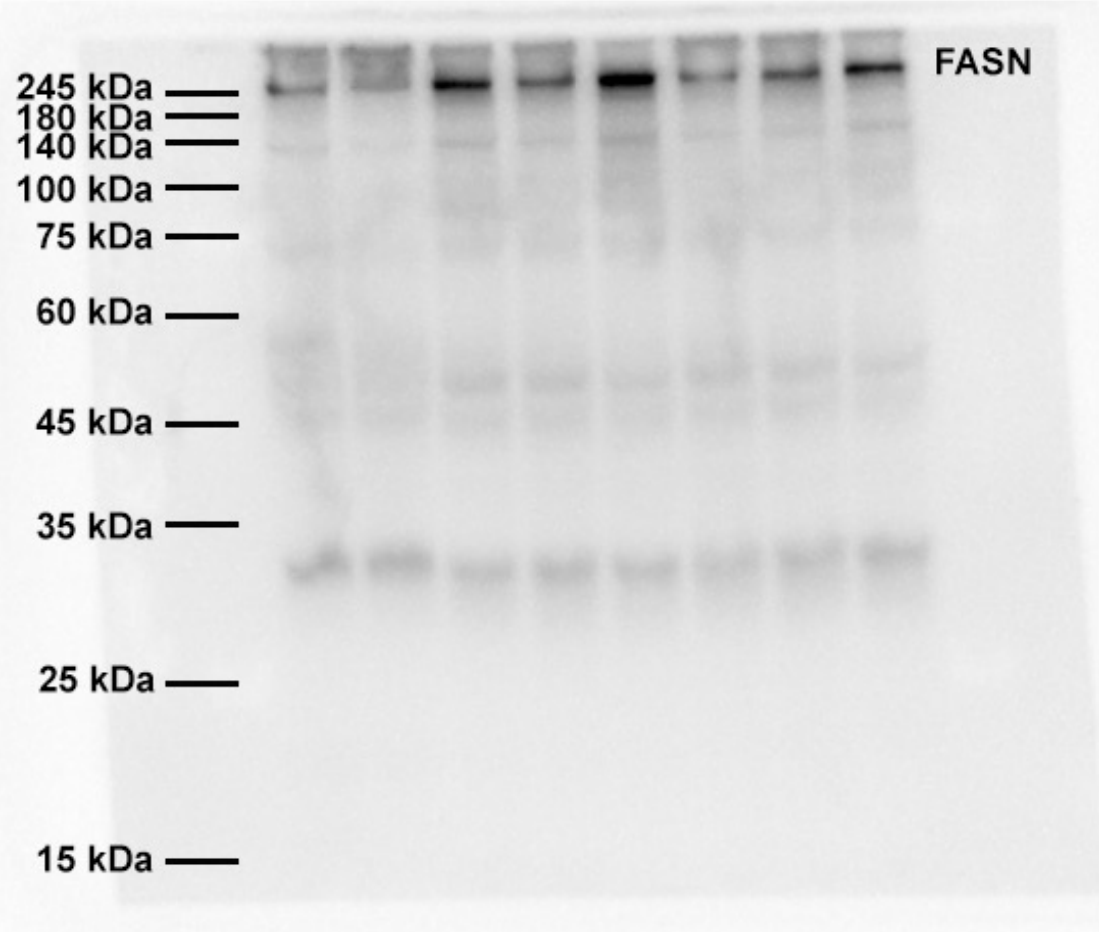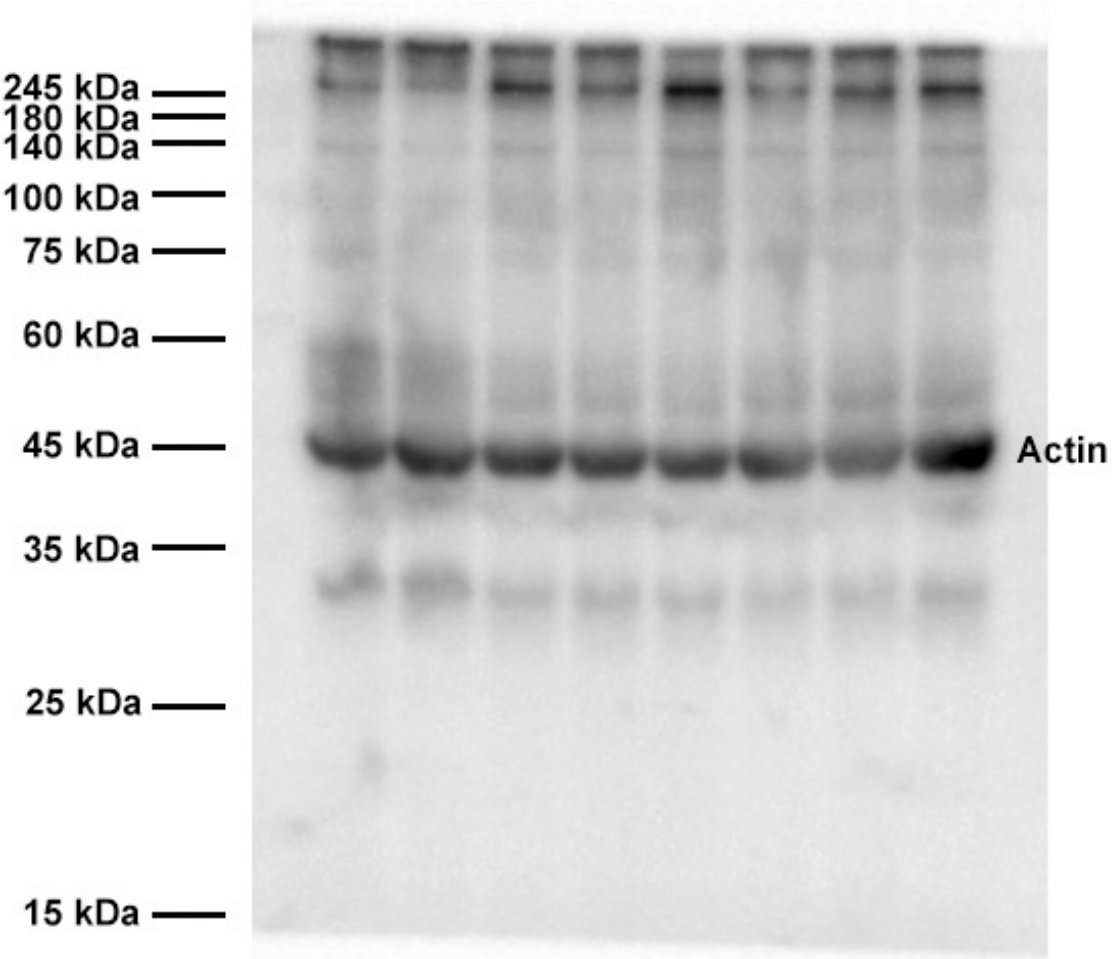

Figure 2B

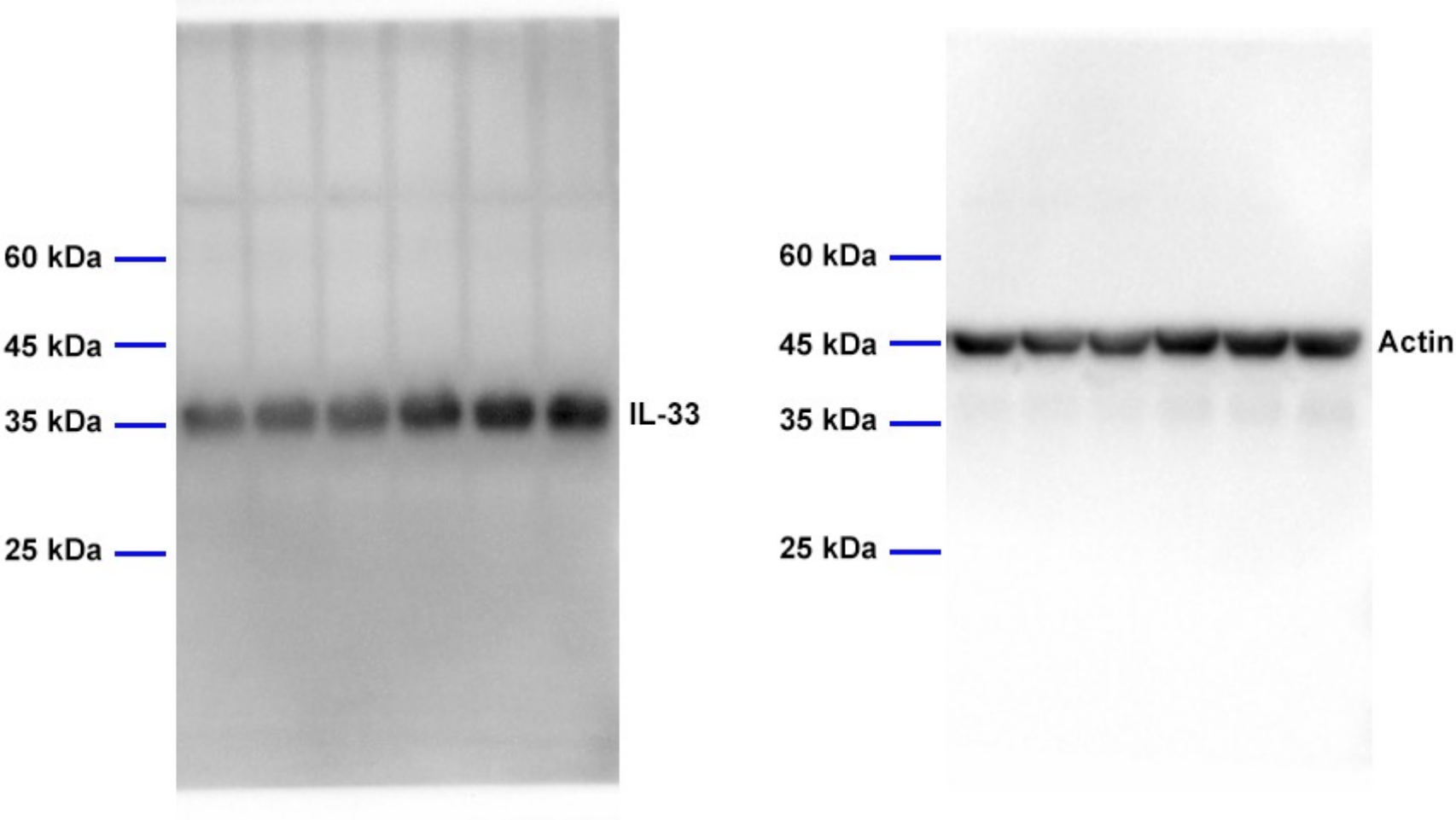

Figure 3F

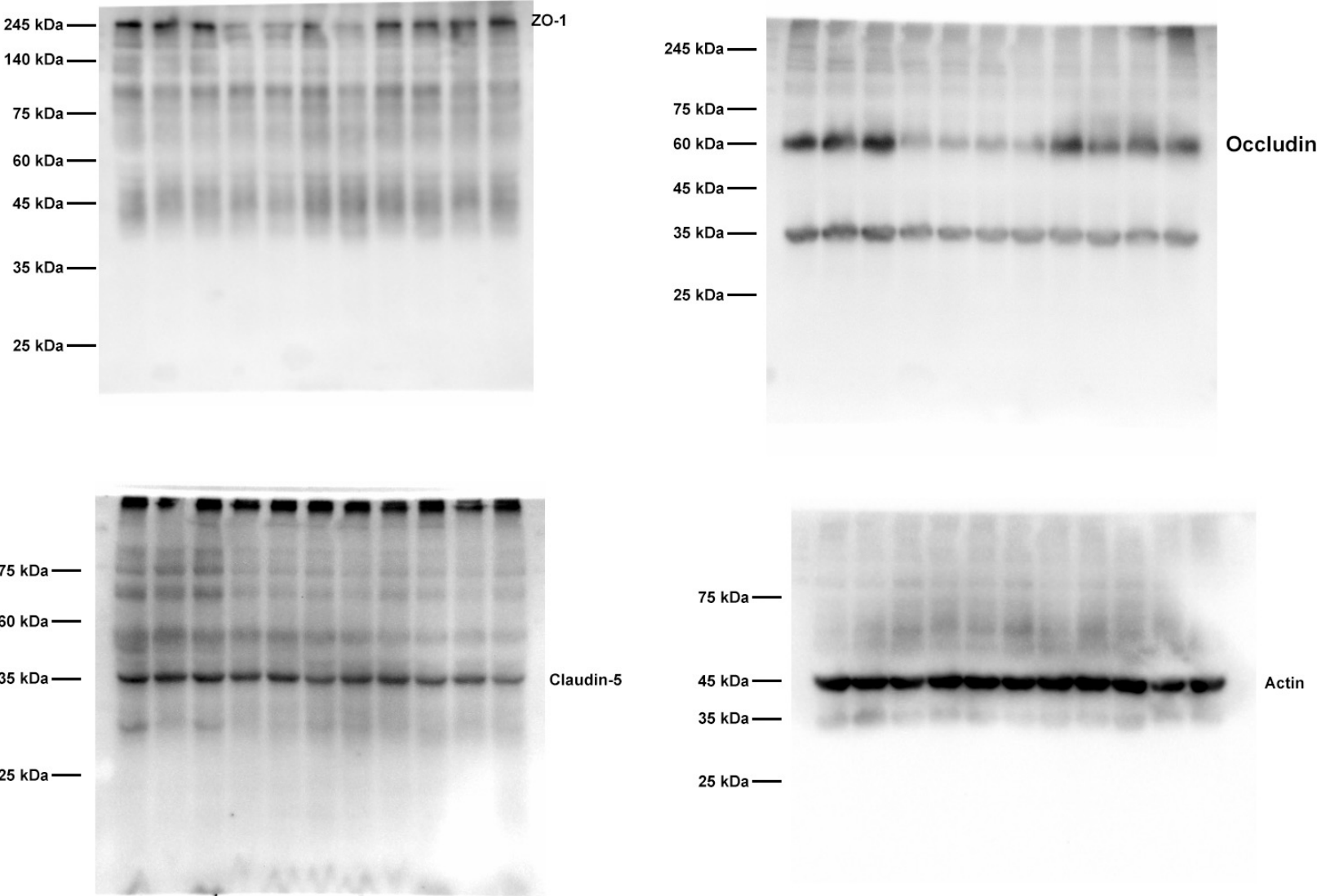

Figure 5A

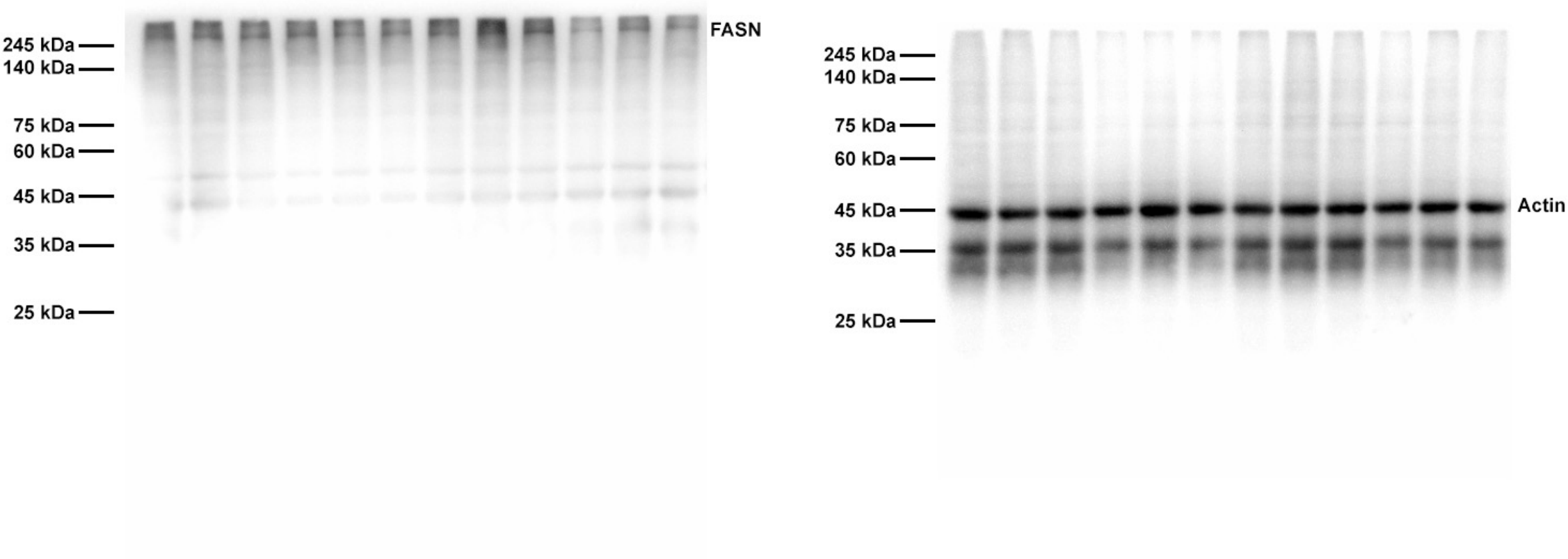

Figure 6B

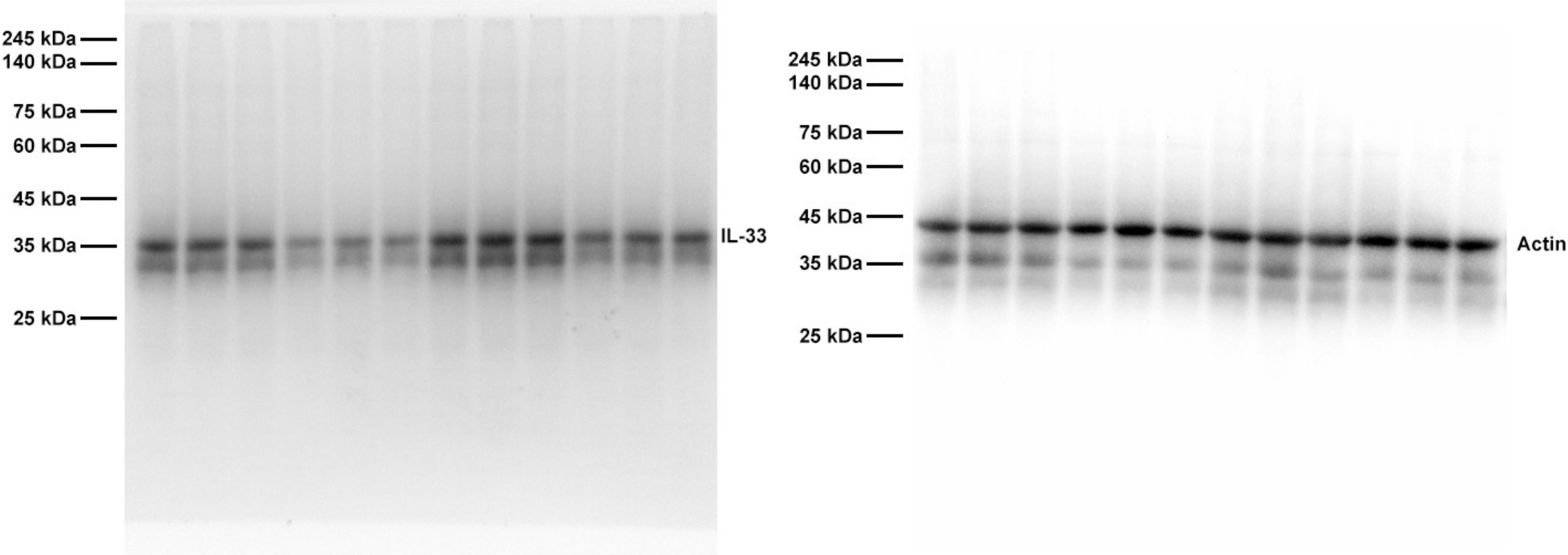

Figure 7F

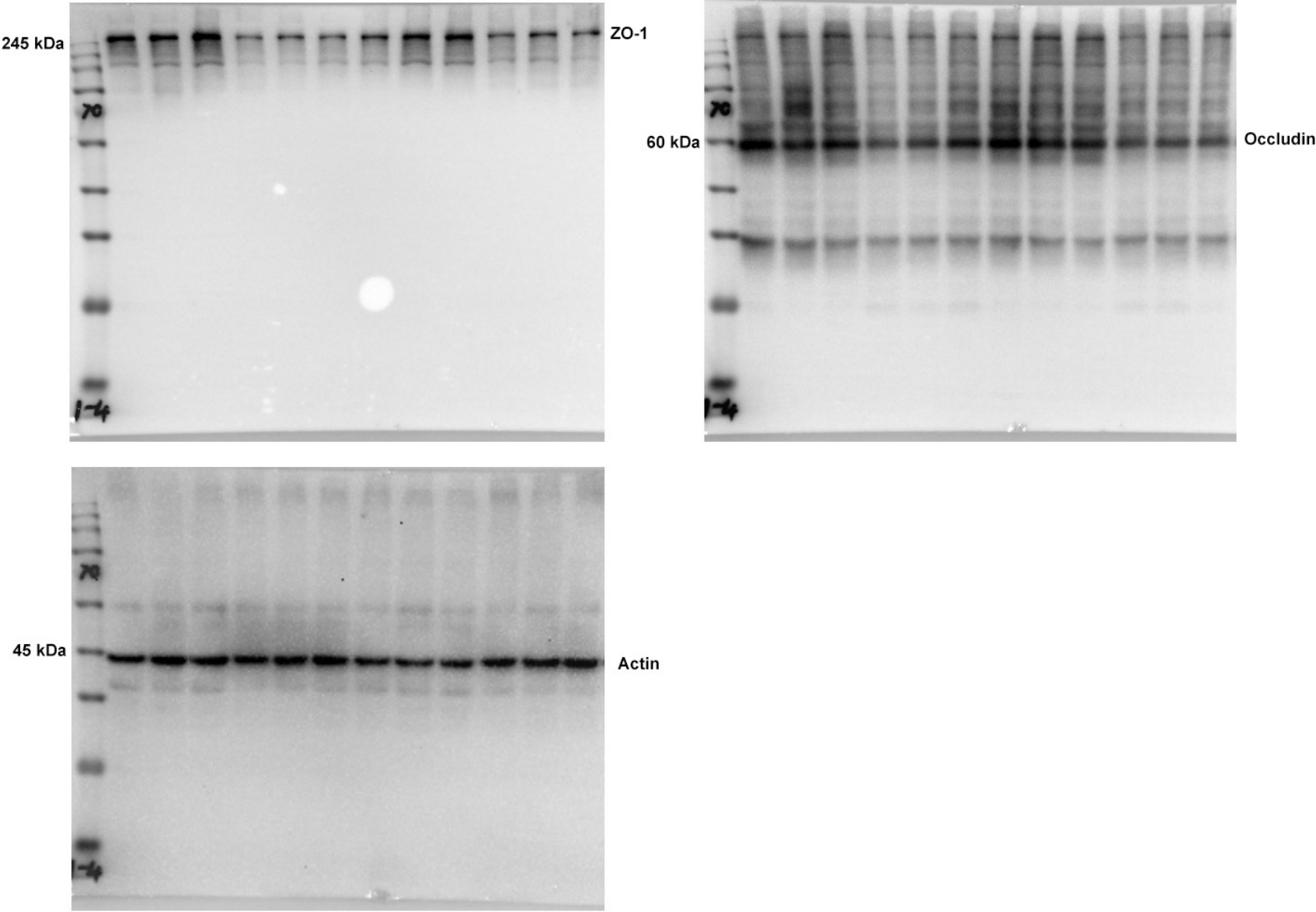

Figure 9F

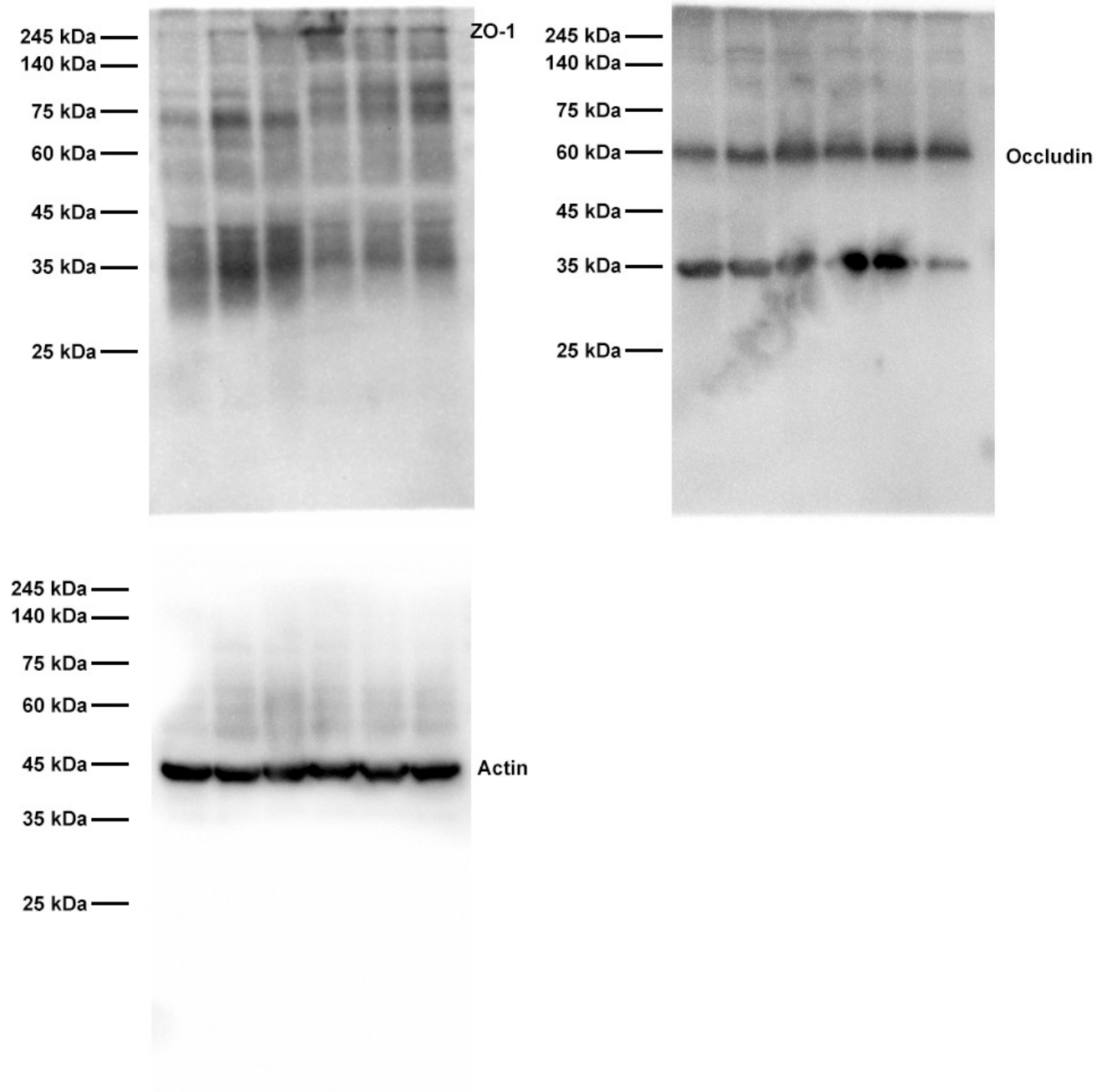

Supplementary figure

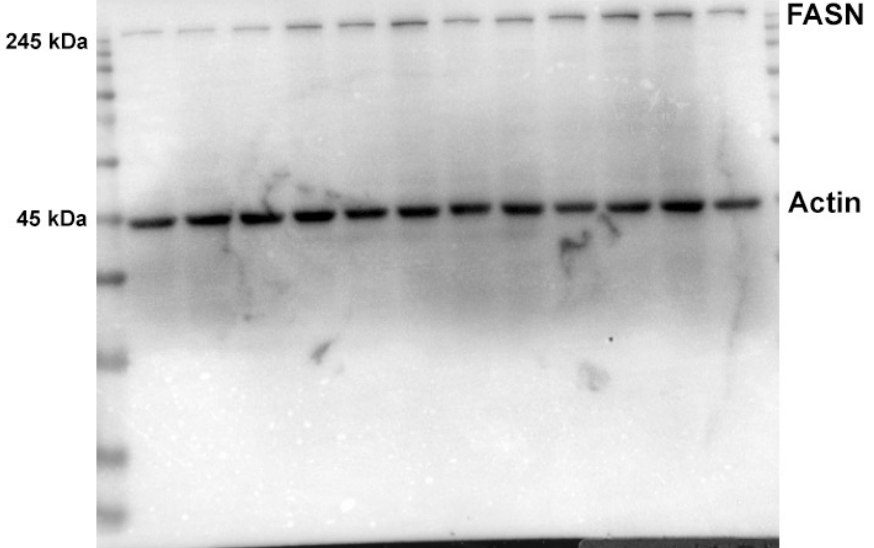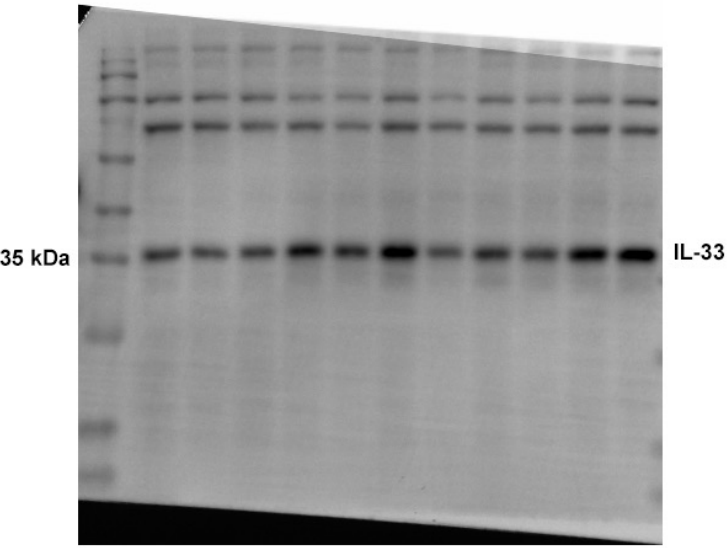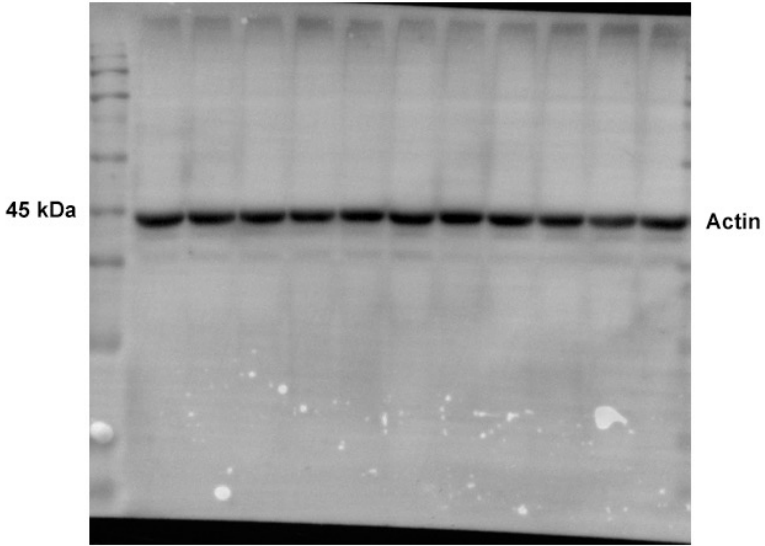

Supplement: Supplementary file 2 — Additional file 2. The uncropped gel and blot images. [file 12974_2023_2942_MOESM2_ESM.pdf]
